# Cell-cycle dependent nuclear gene delivery enhances the effects of E-cadherin against tumor invasion and metastasis

**DOI:** 10.1038/s41392-023-01398-4

**Published:** 2023-05-08

**Authors:** Liting Xie, Jieqiong Wang, Liming Song, Tianan Jiang, Fei Yan

**Affiliations:** 1 Department of Ultrasound, The First Affiliated Hospital, Zhejiang University School of Medicine, Hangzhou, 310058 China; 2 CAS Key Laboratory of Quantitative Engineering Biology, Shenzhen Institute of Synthetic Biology, Shenzhen Institute of Advanced Technology, Chinese Academy of Sciences, Shenzhen, 518055 China; 3 Department of Orthopedics, Zhujiang Hospital, Southern Medical University, Guangzhou, 510280 China

**Keywords:** Gene therapy, Metastasis

## Abstract

Gene delivery is the process by which foreign DNA is transferred to host cells, released from intracellular vesicles, and transported to the nuclei for transcription. This process is frequently inefficient and difficult to control spatiotemporally. We developed a gene delivery strategy that uses ultrasound to directly deliver plasmid DNA into nuclei via gas vesicles (GVs)-based intracellular cavitation. pDNA-binding GVs can be taken up by cells and cause intracellular cavitation when exposed to acoustic irradiation and delivering their pDNA payloads into nuclei. Importantly, GVs can remain stable in the cytoplasm in the absence of acoustic irradiation, allowing for temporally controlled nuclear gene delivery. We were able to achieve spatiotemporal control of E-cadherin nuclear gene delivery in this manner, demonstrating its efficacy in tumor invasion and metastasis inhibition. Interestingly, we discovered that nuclear gene delivery of E-cadherin during the G2/M phase of the cell cycle in C6 tumor cells inhibited tumor invasion and metastasis more effectively than during the G1 and S phases. The gene delivery of E-cadherin at the G2/M phase resulted in significantly lower expression of Fam50a, which reduced Fam50a/Runx2 interaction and led to reduced transactivation of MMP13, an important factor for epithelial-mesenchymal transition, as observed in a molecular mechanism assay. Thus, using remote acoustic control of intracellular cavitation of pDNA-GVs, we developed a high spatiotemporally controllable gene delivery strategy and achieved stronger tumor invasion and metastasis inhibition effects by delivering the E-cadherin gene at the G2/M phase.

## Introduction

Gene delivery is a critical process that transfers foreign DNA to host cells for biomedical applications, such as genetic research or gene therapy.^1,2^ In general, this process necessitates the incorporation of DNA into target cells, its release from intracellular vesicles, and transport to the nucleus, where transcription occurs. The design of effective gene delivery systems, such as biological vectors (e.g., virus or bacteria) or chemical carriers (e.g., lipid or nanoparticle), is critical in most cases for the successful delivery of exogenous genes.^3–5^ Non-viral gene carriers are becoming increasingly appealing due to their lower toxicity and immunogenicity when compared to viral vectors.^6^ However, the main limitations of non-viral carriers are low levels of protein expression and poor gene transfection efficiency.^7^ Using cationic lipids, researchers discovered that it is not complex uptake but rather subsequent steps that limit transfection efficiency.^8,9^ Nuclear gene delivery is considered to be the most important process for gene expression.^10,11^ Due to the loss of nanoparticles during intracellular delivery, only one-fourth of the gene-loaded nanoparticles reach the nucleus.^12^ Therefore, it is a significant challenge to allow exogenous DNA to enter the nucleus and augment the efficiency of nonviral vectors without causing side effects. Furthermore, both viral and non-viral strategies lack the capability of on-demand gene delivery in a spatially and temporally controlled manner, which is critical for investigating time- or cell-cycle-dependent gene functions or evaluating the in vivo effect of local gene expression.^13,14^

In the last decade, several types of physically triggered gene delivery strategies, such as electroporation, photoporation, and sonoporation, have emerged.^15^ These methods not only allow for the delivery of a wide range of membrane-impermeable effector molecules into different cell types, but also they are the preferred strategy for difficult-to-transfect cells such as immune cells (e.g., T cells, NK cells, dendritic cells, and macrophages), primary stem cells, and neurons.^15^ Juan C et al. recently developed a light-triggered physical transfection technology for efficient intracellular delivery of functional genetic materials into mammalian cells by rapidly heating the nanobombs and propelling the nanoprojectiles coated with pDNA through the cell membranes of nearby cells, achieving a transfection efficiency of about 20%.^16^ Sonoporation, on the other hand, has clear advantages over other modalities due to its noninvasiveness, high spatiotemporal resolution, and tissue penetration caused by the cavitation of microbubbles caused by ultrasound.^17,18^ Mechanically, when excited by ultrasound at an appropriate frequency and energy, microbubbles can oscillate, grow, and collapse, resulting in a variety of stable or inertial cavitation effects such as microstreaming and microjets.^19–21^ These physical forces cause transient and repairable pores in the cell membrane, allowing foreign substances to enter the cells.^22–24^ In pre-clinical studies, microbubble cavitation-based gene delivery has been shown to be a promising approach and is widely used for the delivery of transgenes for various diseases.^25,26^ Despite this, microbubble-based sonoporation has been plagued by low gene transfection efficiency (approximately 10%).^27^ One reason affecting the microbubble-based sonoporation is the relative distance between the oscillating bubbles and cells.^28^ Another reason may attribute to the extracellular cavitation, which only acts on cell membrane, leading to the low efficient DNA delivery.

**Fig. 1:**
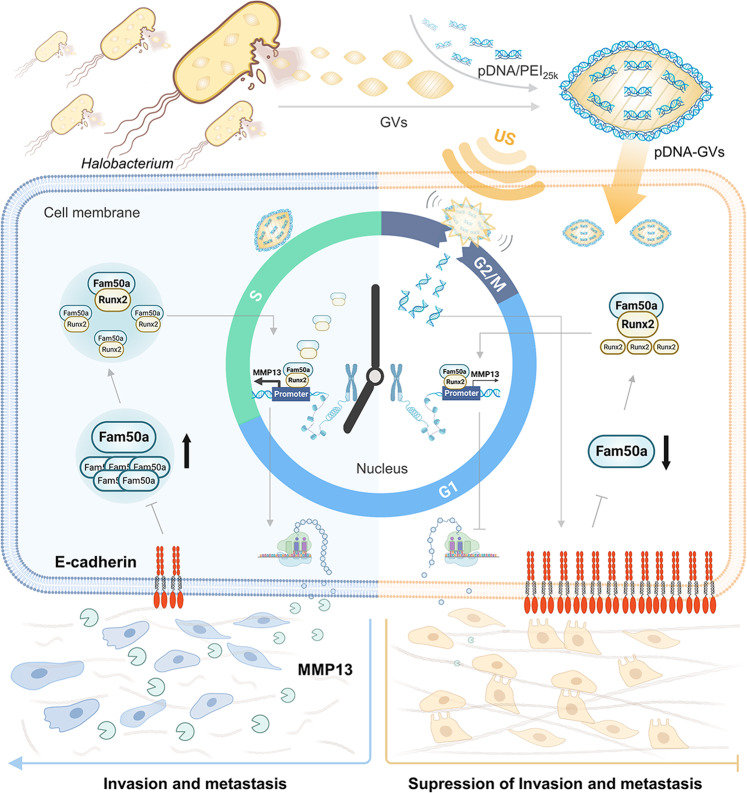
Schematic illustration of nuclear gene delivery via intracellular cavitation of pDNA-GV triggered by acoustic irradiation. pDNA-GV were obtained by electrostatic binding of pDNA/PEI_25 k_ complexes to the surface of negatively charged GVs and were taken up in C6 tumor cells, which have high invasion and metastasis potentials due to the loss of E-cadherin, a key cell adhesion molecule to maintain the epithelial phenotype. Without acoustic irradiation, pDNA-GVs can remain stable in cells due to their intracellular origin and good biocompatibility. When received by acoustic irradiation, these intracellular pDNA-GVs would induce the generation of a series of cavitation effects, perforating the nuclear membrane and delivering their pDNA payloads to the nuclei. The relative stability of GVs in tumor cells makes it possible to trigger pDNA nuclear delivery at different cell cycle phases or to explore the function of transgenes at different cell cycle phases. By this strategy, nuclear delivery of the *E-cadherin* gene was successfully carried out in the G2/M phase, significantly reducing the expression of Fam50a, nuclear translocation of Fam50a/Runx2 and MMP13 transcription, and thus ultimately leading to inhibition of tumor invasion and metastasis. Created with BioRender

Here, we developed gas vesicles (GVs)-based intracellular cavitation to deliver plasmid DNA (pDNA) directly into nuclei via ultrasound in this study. Unlike microbubbles, GVs from *Halobacterium NRC-1* have nanoscale particle size (~200 nm) and can be uptaken by cells via endocytosis, making it possible generate the intracellular cavitation for nuclear gene delivery. We began by isolating GVs from *Halobacterium NRC-1* and coating them with pDNA-polyethylenimine complexes (pDNA/PEI_25 k_). The cells then took up the resulting pDNA/PEI_25 k_-binding GVs (pDNA-GVs). These intracellular pDNA-GVs would generate cavitation effects in the cytoplasm when exposed to ultrasound, perforating nuclear membranes and delivering their pDNA payloads into nuclei. Due to their intracellular origin, GVs can remain stable in cells without ultrasound irradiation (Fig. 1, right panel), giving them significant advantages in temporally controlled nuclear gene delivery. It was surprising that nuclear delivery of the *E-cadherin* gene during the G2/M phase of the cell cycle inhibited tumor invasion and metastasis more effectively than asynchronized cells or cells delivered with acoustic control of gene delivery during the G1 and S phases. The overexpression of E-cadherin during the G2/M phase resulted in the down-regulation of Fam50a, further reducing Fam50a/Runx2 interaction and leading to the down-regulation of MMP13, an important factor for epithelial-mesenchymal transition (EMT) (Fig. 1, left panel).

In this study, we developed an acoustically triggered nuclear gene delivery strategy with high spatiotemporal controllability, providing a useful tool to regulate the behaviors of genetically engineered cells to investigate the underlying mechanisms of genes in vivo.

## Results

### Intracellular cavitation can improve gene transfection efficiency through nuclear gene delivery

**Fig. 2:**
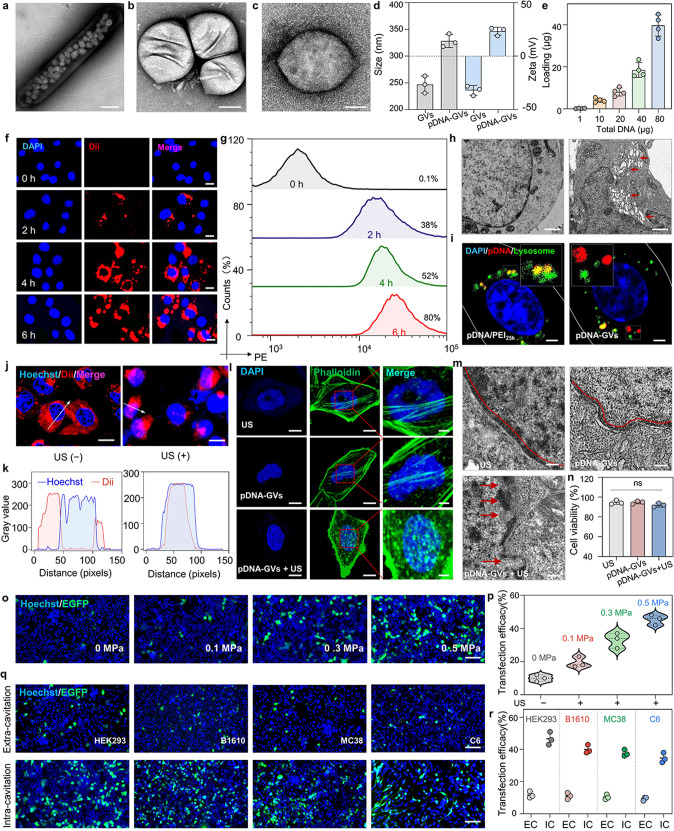
Characterization of pDNA-GVs and nuclear gene delivery by intracellular cavitation. **a** SEM image of *Halobacterium NRC-1* with the GVs in the bacterium. Scale bar = 500 nm. **b** SEM image of the biosynthetic GV. Scale bar = 100 nm. **c** SEM image of plasmid DNA-loaded GV (pDNA-GV). Scale bar = 100 nm. **d** The particle size and zeta potential of GV before and after binding to pDNA/PEI_25 k_ complexes. *n* = 3. **e** The amount of plasmid DNA binding to GVs. *n* = 4. **f**, Fluorescence microscope images of HEK293 cells incubated with fluorescently labeled pDNA-GVs (red) for 0, 2, 4, or 6 h. Nuclei were stained with DAPI (blue). Scale bar = 10 µm. **g** flow cytometry analysis of the intensities of fluorescent signals of HEK293 cells from Fig. 2f. **h** TEM images of HEK293 cells (left), and pDNA-GVs@HEK293 cells (right). Arrows denote pDNA-GVs in the cell. Scale bars = 1 µm. **i**, Confocal microscopy images of pDNA/PEI_25 k_@HEK293 and DNA-GVs@HEK293 cells. Cy3-labeled plasmid DNA was used (red), Lysosomes were stained with Lysotracker (green), and nuclei were stained with DAPI (blue). Scale bar = 2.5 µm. **j** Nuclear delivery of payloads via intracellular cavitation triggered by acoustic irradiation. The pDNA-GVs were labeled with Dii (red). Nuclei were stained with DAPI (blue). Scale bar = 10 µm. **k** Profile histograms indicate the fluorescence intensity for pixels along line scans from the images of Fig. 2j. **l** Fluorescent staining images of HEK293 cells for cytoskeleton with FITC-phalloidin (green) and nuclei with DAPI (blue), showing the messy orientation of the cytoskeleton after intracellular cavitation. Scale bar = 5 µm (left and middle panels), Scale bar = 2 µm (right panel). **m** TEM images of HEK293 cells after intracellular cavitation, showing the destructed nuclear membrane (Red arrows). The nuclear membrane was marked by red lines. Scale bar = 200 nm. **n** Cell viability of the only irradiated HEK293 cells, pDNA-GVs@HEK293 cells, and irradiated pDNA-GVs@HEK293 cells. **o**, Representative fluorescence microscopy images of pEGFP-GVs@HEK293 cells irradiated with different acoustic pressures, revealing a gradually increased EGFP gene transfection efficiency. *n* = 3. Scale bar = 100 µm. **p** The flow cytometry analysis of gene transfection efficiency from Fig. 2o. *n* = 3. **q** Representative fluorescence microscopy images of HEK293 cells, B1610 cells, MC38 cells, and C6 cells treated with intracellular or extracellular cavitation. Scale bar = 100 µm. **r** Flow cytometry analysis of gene transfection efficiency by intracellular or extracellular cavitation. *n* = 3. EC represents extra-cavitation and IC represents intra-cavitation

The GVs were first biosynthesized by *Halobacterium NRC-1* and then isolated, as shown in Supplementary Fig. 1a. The images taken with a scanning electron microscope (SEM) revealed that GVs and pDNA-GVs have a uniform rugby-shaped morphology (Fig. 2a–c). The GVs have a hydrodynamic size of 247.0 ± 16.0 nm and a zeta potential of –32.0 ± 4.6 mV. (Fig. 2d). Surface binding with pDNA/PEI_25 k_ complexes did not alter GV morphology but did reverse their negative zeta potential to positive 23.3 ± 3.8 mV, and slightly increased the particle size of 328.3 ± 12.3 nm (Fig. 2d). The ability to bind DNA gradually increased, reaching nearly 40 µg per OD_500_ GVs (Fig. 2e and Supplementary Fig. 1b). The resulting pDNA-GVs, like GVs, could be stable in PBS with pH values ranging from 5 to 8 for 72 h (Supplementary Fig. 1c). After 6 h of incubation, the pDNA-GVs were efficiently uptaken by HEK293 cells and C6 cells (pDNA-GVs@cells), with cell uptake efficiency of 77.3 ± 2.5% and 85.3 ± 4.5%, respectively (Fig. 2f, g and Supplementary Fig. 2a, b). There was no obvious cytotoxicity observed in these cells after 48 h of incubation with GVs or pDNA-GVs (Supplementary Fig. 3). The cytoplasmic localization of pDNA-GVs surrounding the cell nuclei was confirmed using a transmission electron microscope (TEM) (Fig. 2h). Unlike the pDNA/PEI_25 k_ complex, which travels via the endolysosomal pathway, we discovered that pDNA-GVs could escape the lysosome (Fig. 2i). The reasons may attribute to the relatively large particle size of pDNA-GVs which restricts them to enter lysosomes, and the proton sponge effect of PEI which helps them to escape from lysosomes. Acoustic irradiation of these cells, which contained fluorescently labeled pDNA-GVs, destroyed these GVs and delivered the payload into the nuclei via cavitation effects (Fig. 2j, k). The cytoskeleton was significantly disordered in the irradiated cells but not in the non-irradiated cells (Fig. 2l and Supplementary Fig. 4a), confirming the presence of intracellular cavitation in these irradiated pDNA-GVs@cells. The TEM images showed large holes on the nuclear membrane in these pDNA-GVs@ cells treated with acoustic irradiation, but not in the only irradiated plain cells or the non-irradiated pDNA-GVs@cells (Fig. 2m and Supplementary Fig. 4b). These irradiated pDNA-GVs@cells showed no significant damage to cell viability (Fig. 2n). After intracellular cavitation of pDNA-GVs carrying pCMV-IRES-EGFP plasmid (pEGFP-GVs) in an acoustic intensity-dependent manner, 45.3 ± 3.1% gene transfection efficiency for HEK293 cells was achieved at 0.5 MPa acoustic pressure for 1 min (Fig. 2o, p). In the pEGFP-GVs@cells without acoustic irradiation, only 10.0 ± 2.0% of cells emitted green fluorescence. To determine whether intracellular cavitation outperformed extracellular cavitation, we incubated pEGFP-GVs at the same dose with HEK293 cells, B1610 cells, MC38 cells, and C6 cells, followed by 0.5 MPa acoustic irradiation. Compared to the cells treated with extracellular cavitation, all four cells treated with intracellular cavitation was significantly higher the gene transfection efficiency, with 46.7 ± 4.0% vs. 11.7 ± 2.1% for HEK293 cells, 40.0 ± 2.6% vs. 11.0 ± 2.0% for B1610 cells, 37.7 ± 2.1% vs. 10.3 ± 1.5% for MC38 cells and 34.7 ± 3.1% vs. 9.3 ± 1.2% for C6 cells (Fig. 2q, r). These results suggested that acoustic control of nuclear gene delivery via pDNA-GV intracellular cavitation can significantly improve gene transfection efficiency.

### Temporal control of nuclear *E-cadherin* delivery inhibits the invasion and migration of tumor cells in vitro

**Fig. 3:**
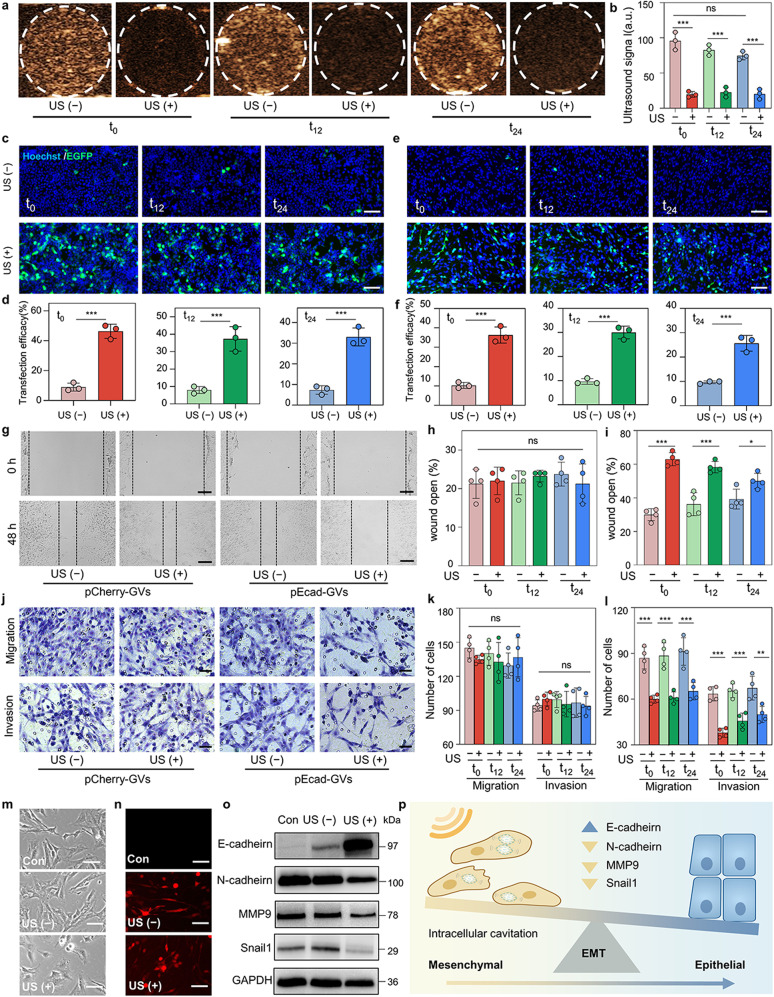
Temporal control of nuclear gene delivery of *E-cadherin* inhibits the migration and invasion of tumor cells. **a** Ultrasound contrast images of pDNA-GVs@HEK293 cells before and after acoustic irradiation at t_0_, t_12_, or t_24_, revealing that the GVs in the cells could be destructed in a temporally controllable manner. The contrast signals disappeared upon the destruction of the GVs. Here, t_0_, t_12_ or t_24_ denote the waiting time after uptake of pDNA-GVs by HEK293 cells. **b** Quantification analysis of the intensities of ultrasound signal for Fig. 3a. *n* = 3. ***P < 0.001. **c** Representative fluorescence microscopy images of EGFP expression through acoustic control of nuclear gene delivery in pEGFP-GVs@HEK293 cells at t_0_, t_12_ or t_24_. Scale bar = 100 µm. **d** Quantification analysis of Fig. 3c by flow cytometry. *n* = 3. ***P < 0.001. **e**, Representative fluor**e**scence microscopy images of EGFP expression through acoustic control of nuclear gene delivery in pEGFP-GVs@C6 cells at t_0_, t_12_, or t_24_. Scale bar = 100 µm. n = 3. **f** Quantification analysis of Fig. 3e. *n* = 3. ***P < 0.001. **g** Representative bright images of the wound healing assay for pCherry**-**GVs@C6 cells or pEcad**-**GVs@C6 cells received with or without nuclear gene delivery at t_0_. Black dotted lines marked the boundaries of tumor cells. Scale bar = 100 µm. **h, i** Quantitative analysis of the wound healing assay for pCherry-GVs@C6 cells (h) and pEcad-GVs@C6 cells (**i**) treated with or without acoustic irradiation treatment at t_0_, t_12_ or t_24_. *n* = 4. *P < 0.05, ***P < 0.001. **j** Microscopy images of migrated or invaded pCherry-GVs@C6 cells and pEcad-GVs@C6 cells treated with or without acoustic irradiation at t_0_. Scale bar = 25 µm. **k, l** Quantitative analysis of Transwell migration and invasion assays for pCherry-GVs@C6 cells (k) and pEcad-GVs@C6 cells (**l**) treated with or without acoustic irradiation at t_0_, t_12_ or t_24_. *n* = 4. **P < 0.01, ***P < 0.001. **m, n** Cell morphology and fluorescence images of plain C6 cells or pEcad-GVs@C6 cells treated with or without intracellular cavitation-mediated overexpression of E-cadherin after 7 days. Scale bar = 10 µm. **o** Western blot analysis of EMT markers, including E-cadherin, N-cadherin, MMP9 and Sanil1 in plain C6 cells and irradiated or non-irradiated pEcad-GVs@C6 cells. **p** Schematic illustration of intracellular cavitation-mediated *E-cadherin* delivery that reverses the EMT process of C6 tumor cells. Created with BioRender

Because the GVs are produced by bacterial cells and can be used as ultrasound contrast agents, we assessed the stability of the pDNA-GVs in cultured mammalian cells. To put it to the test, pDNA-GVs@HEK293 cells were imaged by ultrasound at 0 h (t_0_), 12 h (t_12_), and 24 h (t_24_) after pDNA-GVs uptake. According to Fig. 3a, all these pDNA-GVs@HEK293 cells produced strong contrast signals at t_0_, t_12_, and t_24_. Due to the destruction of GVs, a short high-power acoustic burst could destroy these imaging signals. Notably, only minor signal decay occurred from 0 to 24 h without a high-power burst (Fig. 3b), confirming that these cellular pDNA-GVs can exist in cells for 24 h. The feasibility of temporal control of nuclear gene delivery via intracellular cavitation was then investigated further. After incubating HEK293 and C6 cells with pEGFP-GVs for 6 h, the resulting pEGFP-GVs@HEK293 and pEGFP-GVs@C6 cells were exposed to acoustic irradiation for 1 min at t_0_, t_12_, or t_24_. Figure 3c clearly demonstrated that a large number of EGFP-expressing cells were present in these pEGFP-GVs@HEK293 cells after acoustic irradiation, with 46.3 ± 4.7%, 37.3 ± 7.0%, and 33.0 ± 4.4%. Cells expressing % EGFP for t_0_, t_12_, and t_24_, respectively (Fig. 3d). In contrast, only 9.0 ± 2.6%, 8.0 ± 2.0%, 7.3 ± 2.1% EGFP-expressing cells could be found in these cells, which did not receive acoustic irradiation at t_0_, t_12_ or t_24_. A similar trend was also seen in the pEGFP-GVs@C6 cells, with 36.3 ± 4.2% vs 10.3 ± 1.5%, 30.0 ± 2.6% vs 9.7 ± 1.2% and 25.7 ± 3.2% vs 9.7 ± 0.6%, EGFP-expressing cells for acoustic irradiation vs non-irradiation at t_0_, t_12_ and t_24_, respectively (Fig. 3e, f).

To investigate the availability of intracellular cavitation-mediated nuclear gene delivery for other functional genes, we created pCMV-IRES-mCherry (pCherry) or pCMV-E-cadherin-IRES-mCherry (pEcad) plasmids and tested their effects on invasion and migration of C6 tumor cells via intracellular cavitation in a time-controlled manner (Supplementary Fig. 5a). For 6 h, C6 cells were used to uptake pCherry-GVs or pEcad-GVs to produce pCherry-GVs@C6 cells or pEcad-GVs@C6 cells. The results of fluorescence microscopy confirmed that the mCherry and E-cadherin genes were successfully expressed after acoustic irradiation (Supplementary Fig. 5b, c). The wound healing assay was then used to investigate the migration behaviors of pCherry-GVs@C6 cells and pEcad-GVs@C6 cells with and without acoustic irradiation at t_0_, t_12_, and t_24_. The wound open area did not differ significantly between irradiated pCherry-GVs@C6 cells and non-irradiated pCherry-GVs@C6 cells, as expected. In contrast, pEcad-GVs@C6 cells exposed to acoustic irradiation had significantly higher wound open area at all three-time points when compared to non-irradiated pEcad-GVs@C6 cells (Fig. 3g–i and Supplementary Fig. 6). The results of Transwell assay data also revealed that acoustic irradiation at t_0_, t_12_, and t_24_ reduced the number of pEcad-GVs@C6 cells that migrated and invaded into the bottom chamber in comparison to the number of non-irradiated pEcad-GVs@C6 cells or irradiated pCherry-GVs@C6 cells (Fig. 3j–l and Supplementary Fig. 7). These findings suggest that temporal control of intracellular cavitation-mediated nuclear *E-cadherin* delivery may inhibit C6 tumor cell invasion and migration.

Numerous studies have shown that increasing E-cadherin expression can reverse EMT, a cellular program critical for tumor malignant progression that is characterized by the loss of epithelial phenotype and the acquisition of mesenchymal phenotype.^29–31^ Therefore, we analyzed how cell morphology changed in irradiated and non-irradiated pEcad-GVs@C6 cells. Figure 3 m-n clearly showed that the irradiated pEcad-GVs@C6 group had significantly more cuboidal-shaped cells than the non-irradiated pEcad-GVs@C6 group. We performed a western blotting analysis with some key markers related to EMT to further demonstrate the successful reverse of EMT. Compared to plain C6 cells and non-irradiated pEcad-GVs@C6 cells, irradiated pEcad-GVs@C6 cells had significantly lower expression levels of N-cadherin, MMP9, and Sanil1(mesenchymal markers), all of which play important roles in tumor cell migration and invasion (Fig. 3o). Thus, when pEcad-GVs@C6 cells were exposed to ultrasound, intracellular cavitation occurred, resulting in on-demand overexpression of E-cadherin (epithelial marker) and down-regulation of N-cadherin, MMP9, and Sanil1 (mesenchymal markers). These changes contribute to the reversal of the EMT process, thus inhibiting tumor cell migration and invasion (Fig. 3p).

### Spatiotemporal control of nuclear *E-cadherin* delivery inhibits tumor invasion and metastasis in vivo

**Fig. 4:**
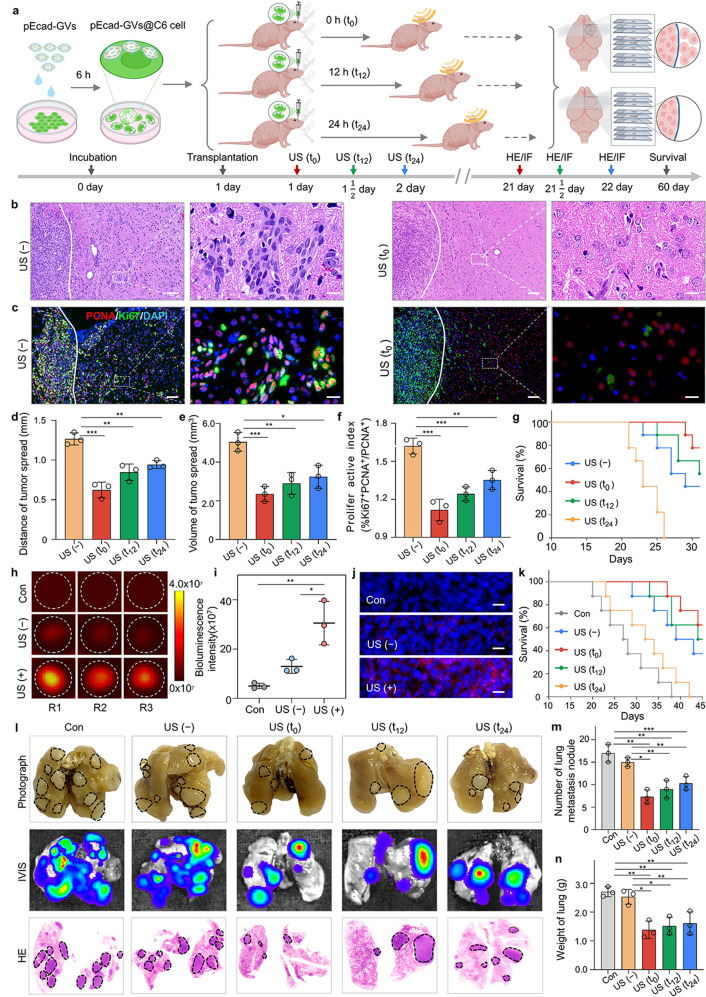
Intracellular cavitation-mediated E-cadherin delivery inhibited tumor invasion and metastasis in vivo. **a** Schematic illustration of nuclear *E-cadherin* gene delivery triggered by acoustic irradiation in the orthotopically transplanted C6 tumor model. Created with BioRender. **b** Representative H&E staining images of brain tumor sections of mice with t_0_-, t_12_, or t_24_-irradiated or nonirradiated tumor. The boundaries of the main tumor mass were marked by white lines. Scale bar = 100 µm. Local peritumoral regions with equal distances away from the boundaries of the main tumor mass were enlarged in the right panels. Scale bar = 20 µm. **c** Representative immunofluorescent staining images of brain tumor sections stained with DAPI (blue), anti-PCNA antibody (red) and anti-Ki67 antibody (green) for US(-) and US(t_0_) group. Scale bar = 100 µm. The local enlarged images were from peritumoral regions with equal distances away from the boundaries of the main tumor mass (right panel). Scale bar = 20 µm. **d** Quantification of tumor cell invasion distances at 21 days after tumor transplantation. *n* = 3. **P < 0.01, ***P < 0.001. **e** Quantification of tumor cell invasion volume at 21 days post-tumor transplantation. *n* = 3. *P < 0.05 **P < 0.01, ***P < 0.001. **f** Quantification of the proliferative index of tumor cells in the peritumoral regions at 21 days after tumor transplantation. *n* = 3. **P < 0.01, ***P < 0.001. **g** Kaplan-Meier survival plot of mice carrying tumors of C6 glioma that received or did not receive acoustic irradiation at t_0_, t_12_ or t_24_ after orthotopic transplantation. *n* = 9. **h, i** Fluorescence images (**h**) and quantitative analysis (**i**) of mCherry expression measured by IVIS in tumors received with or without acoustic irradiation at t_0_. Wild-type C6 tumor cells were used as a control (Con). *n* = 3. *P < 0.05, **P < _0_.01. **j** Fluorescence images of histological sections of tumors received with or without acoustic irradiation. The nuclei were stained with DAPI. Scale bar = 25 µm. **k** Kaplan-Meier survival plot of rats carrying C6 glioma tumors received with or without acoustic irradiation at t_0_, t_12_ or t_24_ after subcutaneous transplantation. *n* = 8. **l**, Representative photographs, IVIS images, and H&E staining images of lung tissues from these treated rats. Three rats from each group were sacrificed on the 35th day after transplantation. *n* = 3. **m**, Number of metastatic lung nodules from each group. *n* = 3. *P < 0.05, **P < 0.01, ***P < 0.001. **n** The weight of the lungs from each group. *n* = 3. *P < 0.05, **P < 0.01

We investigated whether intracellular cavitation-mediated nuclear E-cadherin delivery can inhibit in vivo tumor invasion in a spatiotemporally controllable manner because the acoustic wave, especially at low frequency, can be easily focused and penetrate the deep-seated tumor. To put it to the test, we orthotopically implanted these pEcad-GVs@C6 cells into the brains of mice at t_0_, t_12_, or t_24_, and then irradiated them with ultrasound for 1 min (Fig. 4a, Supplementary Fig. 8). The control cells were pEcad-GVs@C6 cells (transplanted at t_0_, t_12_, or t_24_) that did not receive acoustic irradiation. After 21 days, the brains of three mice from each group were removed, and histological coronal sections of the brain were performed, as shown in the right panel of Fig. 4a. H&E staining sections revealed that tumors treated with acoustic irradiation at t_0_, t_12_, or t_24_ had significantly less invasive potential than non-irradiated tumors that had invaded into the surrounding normal brain tissue (Fig. 4b, Supplementary Fig. 9a). The mean invasion distances of tumor cells from in situ tumors irradiated at t_0_, t_12_, or t_24_ were 0.6 ± 0.1 mm, 0.8 ± 0.1 mm, or 0.9 ± 0.0 mm, respectively, according to quantitative analysis. In contrast, the mean invasion distances achieved by tumors that did not receive irradiation were 1.3 ± 0.1 mm (Fig. 4d). The three-dimensional infiltrative tumor volume calculated from tumor sections was found to be smaller in t_0_-, t_12_-, or t_24_-irradiated tumors than in non-irradiated tumors (Fig. 4e). Furthermore, significantly fewer Ki67^+^/PCNA^+^ tumor cells were found in the surrounding brain tissues of the t_0_-, t_12_-, or t_24_-irradiated tumors than in the nonirradiated tumors, indicating that these tumor cells have a lower proliferation potential away from the irradiated in situ tumors (Fig. 4c and f and Supplementary Fig. 9b). Notably, Kaplan-Meier survival analysis revealed that tumor-bearing mice given acoustic irradiation at t_0_, t_12_, or t_24_ had longer survival times than non-irradiated mice significantly (Fig. 4g). In the orthotopically transplanted glioma model, our findings confirmed the in vivo invasion inhibition effect of intracellular cavitation-mediated nuclear *E-cadherin* delivery.

We next investigated the effects of the intracellular cavitation-mediated nuclear E-*cadherin* delivery on tumor metastasis in vivo. Using pEcad-GVs@C6 cells, a subcutaneously transplanted glioma model was established in newborn Wistar rats. These tumors were given acoustic irradiation for 1 min after 0 h, 12 h, or 24 h post-transplantation (Supplementary Fig. 10). IVIS detected significantly enhanced fluorescence signals in the irradiated tumors (Fig. 4h, i). In contrast, no or only faint mCherry signals were detected in the control and non-irradiated tumors, confirming the successful delivery and expression of nuclear genes after acoustic irradiation. Similarly, histological sections of tumors supported this finding (Fig. 4j). The Kaplan-Meier survival curves of these tumor-bearing rats revealed no significant difference between the nonirradiated pEcad-GVs@C6 group and the plain C6 group, whereas the t_0_, t_12_, or t_24_-irradiated pEcad-GVs@C6 groups showed the significantly higher survival rate, with 46.1 ± 5.8, 44.0 ± 7.0, and 41.6 ± 8.3 days of mean survival time in comparison to the nonirradiated pEcad-GVs@C6 group (32.4 ± 6.9 days) and wild type control group (28.1 ± 6.3 days) (Fig. 4k). Notably, significantly fewer lung metastatic nodules and low lung weight were also noted in the t_0_, t_12_ or t_24_-irradiated pEcad-GVs@C6 groups in comparison to the nonirradiated group and wild-type control group (Fig. 4l–n). All these findings imply that acoustically triggered nuclear *E-cadherin* delivery via intracellular cavitation can inhibit C6 tumor invasion and metastasis in vivo in a spatiotemporal controllable manner.

### Cell cycle-dependent of nuclear *E-cadherin* delivery at the G2/M phase enhances its effects against tumor metastasis

**Fig. 5:**
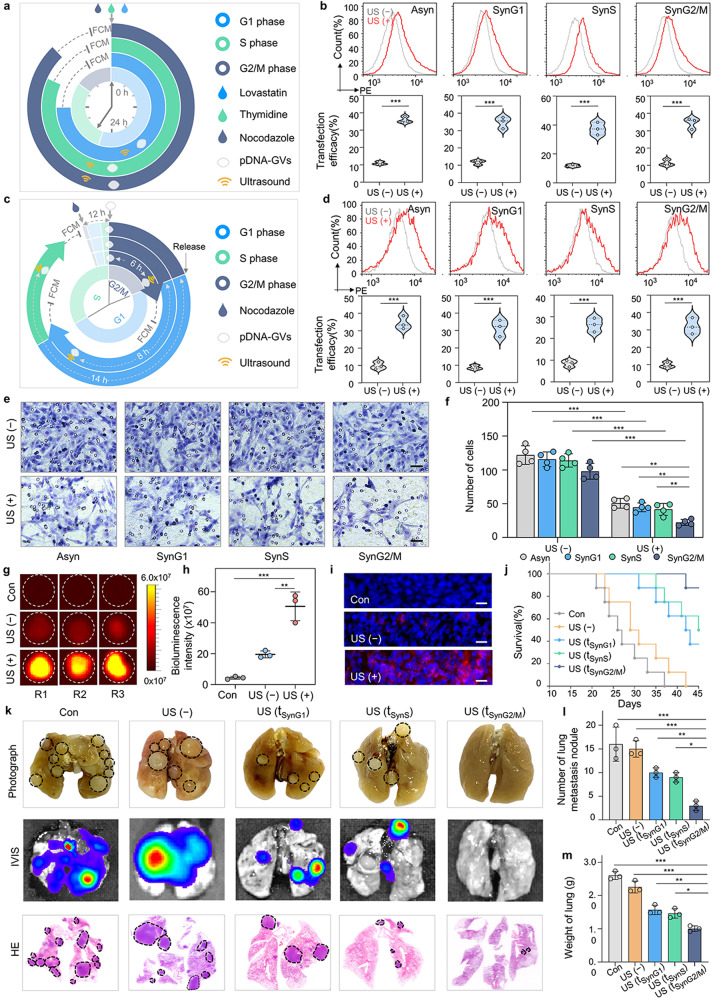
Cell cycle-dependent nuclear gene delivery via intracellular cavitation and its effects of E-cadherin against tumor invasion and metastasis. **a** Schematic illustration of nuclear gene delivery in Asynchronized, or lovastatin-, thymidine, and nocodazole-synchronized tumor cells in the G1, S or G2/M phases, followed by treatment with or without acoustic irradiation. **b** Flow cytometry assays of mCherry gene expression efficiency in the asynchronized or synchronized pCherry-GVs@C6 cells received with acoustic irradiation. *n* = 3. ***P < 0.001. **c** Schemati**c** illustration of nuclear gene delivery in a running cell cycle model, in which nocodazole-synchronized pEcad-GVs@C6 cells are released, followed by nocodazole-free medium release and acoustic irradiation at the SynG2/M, SynG1 or SynS phases. Synchronized pEcad-GVs@C6 were obtained by treating C6 cells for 12 h with nocodazole and then adding pEcad-GVs for cell uptake for 6 h. The cell cycle inhibitor was removed after obtaining synchronized pEcad-GVs@C6 cells. **d** Flow cytometry assays of mCherry gene expression efficiency in the asynchronized or synchronized pEcad-GVs@C6 tumor cells received with or without acoustic irradiation at SynG2/M (0 h after cell cycle release), SynG1 (8 h after cell cycle release), or SynS phases (14 h after cell cycle release) in the operating cell cycle. *n* = 3. ***P < 0.001. **e** Representative imag**e**s of the Transwell assay for asynchronized or synchronized pEcad-GVs@C6 cells treated with or without acoustic irradiation. Ultrasound was applied to the asynchronized pEcad-GVs@C6 cells and synchronized pEcad-GVs@C6 cells in the SynG2/M, SynG1, or SynS phases of the cell cycle. Scale bar = 25 µm. **f** The quantitative results of Fig. 5e, revealing a stronger inhibitory effect of tumor cell migration when nuclear *E-cadherin* delivery was applied in the G2/M phase. *n* = 4. **P < 0.01, ***P < 0.001. **g, h** IVIS images (**b**) and quantitative analysis (**c**) of mCherry expression in tumors received with or without acoustic irradiation in the G2/M phase. Plain C6 cells were used as the control group. *n* = 3. **P < 0.01, ***P < 0.001. **i** Fluorescence images of histological sections of tumors received with or without acoustic irradiation. Nuclei were stained with DAPI. Scale bar = 25 µm. **j** Kaplan-Meier survival plot of Wistar rats bearing tumors from glioma pEcad-GVs@C6 treated with or without acoustic irradiation in the SynG2/M, SynS or SynG1 phases after subcutaneous transplantation. Wild-type C6 cells were used as a control. *n* = 8. **k**, Representative photographs, IVIS images, and H&E staining images of lung tissues from these treated rats. *n* = 3. **l** Number of lung metastatic nodules from each group. *n* = 3. *P < 0.05, **P < 0.01, ***P < 0.001. **m**, The weight of the lungs from each group. *n* = 3. *P < 0.05, **P < 0.01, ***P < 0.001

The cell cycle is a fundamental process required for cell growth and division that is governed by the spatiotemporal regulation of gene expression and protein function.^32,33^ Because E-cadherin expression can be spatiotemporally controlled by ultrasound-triggered intracellular cavitation for nuclear gene delivery, we wonder what effects its overexpression would have on tumor invasion and metastasis if it occurred at different cell cycle phases. Cell cycle synchronization was demonstrated schematically in Fig. 5a by culturing C6 cells in media containing 20 µM lovastatin for the G1 phase, 5 mM thymidine for S phase, and 100 nM nocodazole for the G2/M phase. Flow cytometry analysis revealed that these cells were successfully synchronized, with 80.5 ± 0.7% cells in G1, 91.5 ± 2.1% cells in S, and 92.0 ± 1.4% cells in G2/M. (Supplementary Fig. 11). Next, pCherry-GVs were added to the media for cell uptake in order to obtain synchronized pCherry-GVs@C6 cells, which were then treated with or without acoustical irradiation after 6 h. Figure 5b clearly showed that mCherry-positive cell ratios were significantly higher in these SynG1-, SynS-, and SynG2/M-irradiated cells than in these non-irradiated cells at their respective cell cycle phases. Notably, there were no statistically significant differences in gene transfection efficiency between irradiated pCherry-GVs@C6 cells in different cell cycle phases and asynchronized (Asyn) cells, implying that acoustical control of nuclear gene delivery in different cell cycle phases may achieve similar gene expression levels. To investigate the feasibility of acoustic control of nuclear gene delivery in a running cell cycle, we synchronized C6 tumor cells with nocodazole for 12 h before incubating them with pEcad-GVs for another 6 h to obtain SynG2/M pEcad-GVs@C6 cells, which were then released with nocodazole-free medium (Fig. 5c and Supplementary Fig. 12). The synchronized pEcad-GVs@C6 cells would enter the cell cycle again. As shown in Fig. 5d, these SynG1-, SynS-, and SynG2/M-irradiated cells had significantly higher gene expression efficiency than their phase-corresponding non-irradiated cells, similar to the Asyn-irradiated vs. non-irradiated control cells. Furthermore, synchronized pEcad-GVs@C6 cells were released into the cell cycle through S and G2/M to achieve similar results when they were treated with nuclear *E-cadherin* delivery via acoustic irradiation at the G2/M phase (Supplementary Fig. 13). These findings suggested that acoustically triggering nuclear gene delivery can be used to control transgene expression and achieve comparable expression levels across cell cycle phases.

We synchronized pEcad-GVs@C6 tumor cells and released them for acoustical irradiation at the G2, S, or G1 phases of the running cell cycle to investigate the effects of E-cadherin overexpression on the in vitro invasion behavior of tumor cells. After 48 h, the irradiated cells or their non-irradiated counterparts were transferred to the Transwell insert, and the number of tumor cells that migrated onto the lower surface of the insert was counted. Figure 5e, f and Supplementary Fig. 14 clearly show that Asyn-, SynG2/M-, SynG1-, and SynS-irradiated tumor cells migrated to the lower surface significantly less than non-irradiated controls. Interestingly, we discovered that acoustic irradiation of pEcad-GVs@C6 cells at the SynG2/M phase inhibited migration significantly more than irradiation at the Asyn, SynG1, and SynS phases. To investigate the in vivo effects of intracellular cavitation-mediated nuclear gene delivery of *E-cadherin* at different cell cycle phases, we synchronized pEcad-GVs@C6 cells with nocodazole at the G2/M phase and subcutaneously transplanted them in newborn rats, followed by acoustic irradiation at 0 h, 8 h, or 14 h post-transplantation, corresponding to SynG2/M, SynG1 or SynS phases in the running cell cycle (Supplementary Fig. 15). The successful gene expression of mCherry after the acoustic triggering of nuclear gene delivery at the SynG2/M phase in tumor-bearing rats was shown in Fig. 5g, h. Similarly, histological sections of tumors supported this finding (Fig. 5i). The acoustic control of nuclear *E-cadherin* delivery significantly prolonged the survival of tumor-bearing rats, achieving 52.3 ± 6.9, 41.8 ± 6.8 and 44.9 ± 6.9 days mean survival time for the SynG2/M-, SynG1-, or SynS-irradiated groups, respectively, compared to the synchronized non-irradiated group and wild type control group (Fig. 5j). It was notable that tumor-bearing rats given nuclear *E-cadherin* delivery during the SynG2/M phase lived longer than rats given gene delivery during the SynG1 or SynS phases. In comparison to the non-irradiated and wild-type control groups, the SynG2/M-, SynG1-, or SynS-irradiated groups had significantly fewer lung metastatic nodules and lung weight (Fig. 5k–m). Surprisingly, almost no lung metastatic nodules were found in tumor-bearing rats given acoustic irradiation during the SynG2/M phase. These findings suggest that acoustic control of intracellular cavitation can achieve cell cycle-dependent nuclear *E-cadherin* delivery while also inhibiting tumor invasion and metastasis in vitro and in vivo.

### Mechanisms of the enhanced inhibition effects against tumor invasion and metastasis for nuclear *E-cadherin* delivery at the G2/M phase

**Fig. 6:**
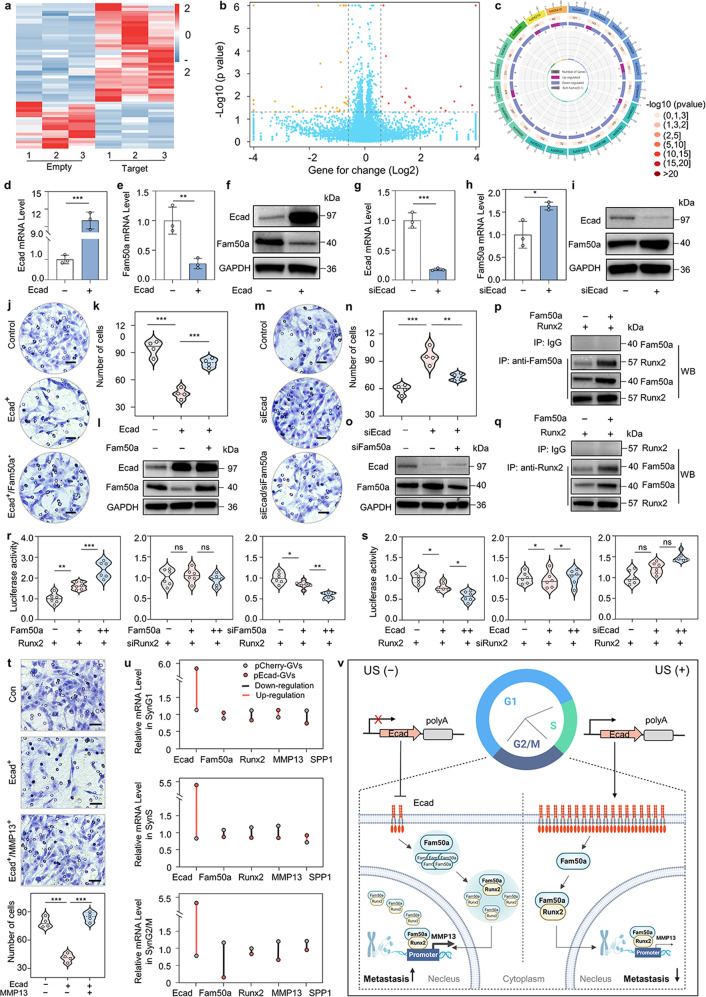
Molecular mechanisms of the enhanced inhibition effects of E-cadherin in the G2/M phase against tumor invasion and metastasis. **a** Heat map of the RNA-seq for pCherry-GVs@C6 cells and pEcad-GVs@C6 cells received with acoustic irradiation in the SynG2/M phase. The mCherry-positive cells were sorted by flow cytometry before RNA-seq. **b** Volcano plot of RNA-seq for C6 tumor cells treated with mCherry or E-cadherin nuclear gene delivery. **c** KEGG enrichment map of RNA-seq for these cells. **d, e** qPCR assays of the transcription levels of Fam50a and E-cadherin in the E-cadherin-overexpressed C6 cells. *n* = 3. **P < 0.01, ***P < 0.001. **f** Western blot assay of the protein levels of Fam50a and E-cadherin in the E-cadherin-overexpressed C6 cells. **g, h** qPCR assays of the transcription levels of Fam50a and E-cadherin in the E-cadherin-knockdown C6 cells. *n* = 3. *P < 0.05, ***P < 0.001. **i**, Western blot assay of the protein levels of Fam50a and E-cadherin in the E-cadherin-knockdown C6 cells. **j** Transwell invasion assay of plain C6 cells (top), E-cadherin-overexpressed C6 cells (middle), and Fam50a- and E-cadherin-overexpressed C6 cells (bottom), showing that the invasion inhibition effect of E-cadherin was reduced by overexpression of Fam50a. Scale bar = 20 µm. **k** The quantitative results of Fig. 6j. *n* = 5. ***P < 0.001. **l** Western blot assay of the expression of E-cadherin and Fam50a in these C6 cells. **m** The Transwell invasion assay of C6 cells (upper), E-cadherin knockdown C6 cells (middle), and Fam50a and E-cadherin knockdown C6 cells (bottom). **n** The quantitative results of Fig. 6m. *n* = 5. **P < 0.01, ***P < 0.001. **o** Western blot assay of the expression of E-cadherin and Fam50a in these C6 cells treated with siFam50a or/and siE-cadherin. **p, q** Co-immunoprecipitation (Co-IP) analysis of protein interaction between Fam50a and Runx2 in Fam50a- and Runx2-overexpressed C6 cells. anti-Fam50a or anti-Runx2 and anti-IgG antibodies were used for Co-IP experiments. **r** MMP13 promoter transcription activity assay in the Runx2-overexpressed or Runx2-knockdown cells transfected with different doses of Fam50a plasmids or siFam50a, showing that MMP13 was the downstream target gene of the Fam50a/Runx2 axis. *n* = 6. *P < 0.05, **P < 0.01, ***P < 0.001. **s** MMP13 promoter transcription activity assay in the Runx2-overexpressed or Runx2-knockdown cells transfected with different doses of E-cadherin plasmids or siE-cadherin. *n* = 6. *P < 0.05. **t** The transwell assay, and quantitative results of C6 cells (top), E-cadherin-overexpressed C6 cells (middle), and E-cadherin and MMP13-overexpressed C6 cells (bottom), showing that MMP13 overexpression could reduce the tumor cell invasion inhibition effect of E-cadherin. *n* = 4. Scale bar = 25 µm. ***P < 0.001. **u** qPCR assays of the transcription levels of E-cadherin, Fam50a, Runx2, MMP13, and SPP1 in synchronized C6 cells received with nuclear *E-cadherin* gene delivery in the G1, S or G2/M phases. **v** Schematic illustration of molecular mechanisms of the enhanced effect of tumor invasion inhibition on C6 tumor cells received with nuclear delivery of the *E-cadherin* gene in the SynG2/M phase. Created with BioRender

We performed RNA sequencing (RNA-seq) of pCherry-GVs@C6 cells and pEcad-GVs@C6 cells treated with acoustic irradiation at the SynG2/M phase to investigate the mechanisms by which overexpression of E-cadherin via acoustically triggered nuclear gene delivery enhanced the inhibition effects against tumor invasion and migration. When comparing tumor cells treated with E-cadherin gene delivery to tumor cells treated with mCherry gene delivery at the SynG2/M phase, researchers discovered 18 up-regulated genes and 38 down-regulated genes (Fig. 6a, b). Pathway and Gene Ontology analysis in the Kyoto Encyclopedia of Genes and Genomes revealed that these differentially expressed genes were significantly enriched in the cellular process, biological adhesion, regulation of biology process, signaling receptor binding, and transcription regulator activity (Fig. 6c and Supplementary Fig. 16). Fam50a was the most clearly down-regulated gene (Fig. 6b). Quantitative PCR (qPCR) and western blotting assays confirmed that Fam50a was significantly down-regulated and E-cadherin was significantly up-regulated in C6 cells treated with *E-cadherin* gene delivery during the SynG2/M phase (Fig. 6d–f). E-cadherin siRNA knockdown revealed a significant increase in Fam50a expression at both the transcription and translation levels (Fig. 6g–i), indicating that E-cadherin may function as a negative regulator of Fam50a. Interestingly, overexpression of Fam50a could reduce the inhibitory effects of E-cadherin against tumor invasion, while knockdown of Fam50a could recover the tumor invasion inhibitory effects for loss of E-cadherin (Fig. 6j–o). These findings suggest that Fam50a inhibits tumor invasion and migration by negatively regulating the function of E-cadherin.

Previous research has shown that Fam50a can interact directly with Runt-related transcription factor 2 (Runx2), a member of the runt homology domain family.^34^ Recent research has discovered that Runx2 plays a critical role in tumor cell invasion and migration.^35–37^ Therefore, we hypothesize that E-cadherin inhibits tumor invasion and metastasis via the Fam50a/Runx2 pathway. To investigate this, we first looked at the protein-protein interaction between Fam50a and Runx2 in C6 cells. Fam50a was co-immunoprecipitated with Runx2 in Fig. 6p-q, confirming that Fam50a can directly interact with Runx2 in tumor cells. As a transcription factor, Runx2 activates several genes involved in invasion and metastasis, including SPP1 and MMP13, in which Fam50a is expected to participate synergistically as a DNA-binding protein.^38,39^ Using an SPP1 or MMP13 promoter-luciferase reporter, we investigated Runx2’s transactivation activity (Supplementary Figs. 17–18). Figure 6r clearly demonstrated that Fam50a successfully activated MMP13 in Runx2-expressed tumor cells but not in Runx2-knockdown tumor cells in a dose-dependent manner. The knockdown of Fam50a decreased the luciferase activity of these cells in Runx2-expressed tumor cells. We also investigated E-transcriptional cadherin’s activation of MMP13(Fig. 6s). The luciferase activity of these cells in the Runx2-expressed tumor cells gradually decreased with the increased expression of E-cadherin, indicating that E-cadherin inhibited MMP13 transactivation in a dose-dependent manner in the Runx2-expressed tumor cells. When Runx2 expression was lost, E-cadherin’s ability to inhibit MMP13 transactivation was lost. Furthermore, knocking out E-cadherin restored the luciferase activity of these cells in Runx2-expressed tumor cells. SPP1 was not activated by Fam50a or E-cadherin in Runx2-expressing tumor cells (Supplementary Fig. 19). All these findings show that Fam50a increases MMP13 transactivation by Runx2, but Fam50a expression is negatively regulated by E-cadherin, resulting in a Fam50a/Runx2-MMP13 axis. We investigated whether the function of E-cadherin against tumor cell migration and invasion via the Fam50a/Runx2-MMP13 axis had any effect on tumor cell invasion. Figure 6t clearly demonstrated that MMP13 overexpression could counteract the tumor invasion inhibition effect of E-cadherin in C6 tumor cells. Given that E-cadherin inhibited tumor cell invasion significantly more effectively in the SynG2/M phase than in the SynG1 and SynS phases, we wonder if its overexpression specifically deactivates the Fam50a/Runx2-MMP13 axis. To test this, we used acoustically triggered nuclear gene delivery at the SynG1, SynS, or SynG2/M phases to overexpress the E-cadherin gene in synchronized C6 tumor cells. The asynchronous tumor cells served as the control. The expression of E-cadherin, Fam50a, Runx2, MMP13, and SPP1 was determined using a qPCR assay. We discovered no significant expression differences for the four genes during the SynG1 and SynS phases, which was consistent with their changes in asynchronized tumor cells (Fig. 6u). Significantly reduced expression of Fam50a and MMP13, but not Runx2 or SPP1, was observed only in the SynG2/M phase of E-cadherin-overexpressing cells. These findings suggested that the enhanced inhibitory effects of E-cadherin overexpression on tumor invasion and migration could be attributed to Fam50a down-regulation during the SynG2/M phase, which would result in reduced nuclear translocation of the Fam50a/Runx2 complexes and decreased the transcription of MMP13 (Fig. 6v).

## Discussion

The regulation of gene expression is a precise process that allows a cell to respond to various physiological, pathological, and environmental stimuli.^40,41^ This process is tightly controlled in a spatiotemporal fashion.^14,16,42^ Therefore, there is a need to develop a tool for temporal and spatial control of gene expression, particularly in vivo. We developed an acoustical control of nuclear gene delivery strategy using pDNA-GVs that can be taken up by cells in this study. GVs have good biocompatibility and can remain stable in recipient cells due to their nanoscale particle size and intracellular origin. They generate intracellular cavitation in response to acoustic irradiation, which can perforate the nuclear membrane and deliver their genetic payloads into the nuclei, resulting in on-demand transcription or expression of transgenes. This nuclear gene delivery strategy clearly has several advantages, including 1) pDNA can be delivered directly into nuclei via intracellular cavitation, allowing for higher gene transfection or expression efficiency; and 2) nuclear gene delivery can be triggered by focused ultrasound in a temporally and spatially controllable manner, providing a promising control strategy for gene expression in vivo.

The advantage of nuclear gene delivery is associated with the GVs’ intracellular origin and small particle size, allowing them to be efficiently uptaken by cells and cause cellular cavitation. Numerous studies have shown that microbubble-based extracellular cavitation only produces about 10% gene transfection efficiency because acoustic cavitation only exerts mechanical forces on the membrane, resulting in cytoplasmic delivery of genetic payloads.^27,28^ Our intracellular cavitation system, on the other hand, may achieve 30–50% gene transfection efficiency and over 90% cell viability in all tested cell types (Fig. 2q), which is significantly higher than previously reported membrane-disrupted gene delivery strategies.^16,43^ Another advantage of spatiotemporally controllable gene delivery is the relative stability of pDNA-GVs in cells, which allows for the cavitation effects to be triggered at a predetermined time for nuclear gene delivery. Furthermore, because ultrasound has a high tissue penetrability and can be focused on specific tissue or cells, on-demand gene delivery can only occur at the local site. It is especially valuable to achieve remote spatiotemporal control of gene expression for tumors or cranial nerve nuclei in vivo.

We used E-cadherin as the transgene in this study and investigated its effects on inhibiting tumor invasion and migration in spatiotemporally controllable gene delivery in vitro and in vivo. As shown in Fig. 3g–l, pEcad-GVs@C6 tumor cells received acoustic irradiation at t_0_, t_12_, or t_24_ and had significantly enhanced inhibition effects when compared to non-irradiated control cells. The cell morphology and molecular biomarker assay revealed EMT reversion in these irradiated tumor cells, confirming E-strong cadherin’s activity as an invasion suppressor. It is also worth noting that we observed E-strong cadherin’s invasion and migration inhibition function in orthotopically transplanted tumor models and subcutaneously transplanted tumor models using pEcad-GVs@C6 tumor cells by acoustic irradiation at different times. To the best of our knowledge, this is the first time that on-demand nuclear gene delivery in vivo has been realized in a spatiotemporally controllable manner.

However, it should be noted that the temporal controllability of nuclear gene delivery can deteriorate over time and eventually lose its superiority over non-irradiated control. The dissociation of the pDNA from the GVs within the cellular condition could be the cause, as their binding only relies on electrostatic interaction. Despite this, we used this system to investigate the role of E-cadherin in the cell cycle. At various stages, cell cycle-dependent nuclear gene delivery was achieved with success. Surprisingly, we discovered that acoustic triggering of nuclear *E-cadherin* delivery at the G2/M phase inhibited tumor invasion and metastasis better in vitro and in vivo than G1 or S phases. The RNA-seq assay revealed that Fam50a was significantly down-regulated in these E-cadherin overexpressed cells during the G2/M phase, resulting in less transcription of MMP13 due to the reduced Fam50a/Runx2 complexes. Numerous studies have shown that MMP13, a matrix metalloproteinase, plays an important role in EMT in many tumors and has a strong correlation with invasive carcinomas.^44,45^ Several studies have found that MMP13 can reduce tumor cell adhesion by degrading the extracellular matrix, thereby facilitating tumor cell invasion and metastasis.^46,47^ Thus, our findings suggest that the increased tumor invasion and migration inhibition effects of E-cadherin during the G2/M phase may be due to the deactivation of the Fam50a/Runx2-MMP13 axis. Given that the G2/M phase is relatively short-lived during a cell cycle, the truth is most likely hidden due to the small cell population of the G2/M phase in the asynchronized tumor cell population. In this context, our research provides a tool for gaining new molecular insights into the cell cycle. In fact, our findings suggest that nuclear delivery of E-cadherin, particularly at the G2/M stage, can more effectively down-regulate Fam50a expression, resulting in less nuclear translocation of the Fam50a/Runx2 complexes and lower transcription of MMP13 compared to G1-, S-phase tumor cells or untreated tumor cells.

In this study, we developed a nuclear gene delivery strategy that can be triggered by remote acoustic irradiation via spatiotemporally controllable intracellular cavitation of pDNA-GVs. Gene transfection efficiency could significantly be increased when compared to conventional microbubble-based extracellular cavitation methods. Using this strategy, the *E-cadherin* gene was investigated for its role in inhibiting tumor invasion and migration. Our in vitro and in vivo results confirmed the feasibility of spatiotemporally controllable acoustical control of nuclear gene delivery. Furthermore, the tumor invasion and migration functions of E-cadherin were studied in different stages of the cell cycle using an acoustic control of nuclear gene delivery strategy. Our findings show that tumor cells received with nuclear gene delivery at the G2/M phase had better tumor invasion and metastasis inhibition effects than those received at the G1, and S phases. Fam50a was significantly downregulated at the G2/M phase in E-cadherin-overexpressed cells, resulting in less transcription of MMP13, according to RNA-seq and molecular mechanism assays. Finally, our findings suggest a novel strategy for achieving spatiotemporally controllable nuclear gene delivery and elucidating the molecular mechanisms of E-cadherin for superior tumor invasion and migration inhibition effects during the G2/M phase. Our strategy might be used for regulating the behaviors of genetically engineered CAR-T cells, CAR-NK cells, or stem cells, as well as for investigating the underlying mechanisms of genes that participate in tumor development and progression in vivo.

## Materials and methods

### Synthesis of GVs and fabrication of pDNA-GVs

Bacterial culture and biosynthetic nanobubble isolation were carried out in accordance with our previous report and Shapiro MG et al. ^48,49^. For two weeks, *Halobacterium NRC-1* (*Halo*) was cultured in ATCC medium at 42 °C with shaking at 100 rpm. These *Halo* were then transferred to a separating funnel and left at room temperature for one week. After floating, the *Halo* cells were separated and lysed with TMC buffer before being centrifuged at 350 g for 4 h. The milky GVs were isolated and stored at 4 °C. PEI_25 k_ (Polysciences Inc) was first incubated with plasmid DNA at an N/*P* = 15 ratio for 30 min to prepare pDNA-GVs. The resulting pDNA/PE_I25 k_ complexes were then incubated with GVs for an additional 40 min to yield pDNA-GVs. Centrifugal flotation was performed to remove the free pDNA/PEI_25 k_ complexes.

### Characterization of GVs and pDNA-GVs

The structure of GVs and pDNA-GVs was analyzed by SEM (Regulus 8100, HITACHI, England). Dynamic light scattering was used to determine the size distribution and zeta potential (Zetasizer Nano ZS, Malvern, UK). A spectrophotometer was used to measure the optical density (OD) at 500 nm (Nanodrop 2000c, Thermo Scientific, USA). The stability of GVs and pDNA-GVs was determined by measuring their size in PBS solution with pH values of 5, 6, 7, or 8 over a period of time (0, 6, 12, 18, or 24 h).

### Preparation and characterization of pDNA-GVs@cells

HEK293 or C6 cells were incubated with pDNA-GVs (pDNA-GVs@ HEK293 cells or pDNA-GVs@ C6 cells) for 2, 4, or 6 h at 37 °C, and free pDNA-GVs was removed via PBS rinse. To evaluate the cellular uptake efficiency of pDNA-GVs, 1,1’-dioctadecyl-3,3,3’,3’-tetramethylindocarbocyanine perchlorate (Dii) dye (Sigma-Aldrich)-labeled pDNA-GVs were incubated with HEK293 or C6 cells, and DAPI (Sigma-Aldrich) was used to stain the pDNA-GVs@HEK293 cells or pDNA-GVs@C6 cells, followed by assay with a confocal microscope (A1, Nikon, Japan) and flow cytometry (CytoFLEX LX, Beckman, USA). TEM was used to examine the morphology and subcellular localization of pDNA-GVs in HEK293 and C6 cells (HT7700, HITACHI, England). To locate pDNA-GVs in lysosomes, pDNA was labeled with N,N’-(dipropyl)-tetramethyl indocarbocyanine (Cy3) using Label IT Nucleic Acid Labeling Kit (Mirus) and following supplier’s protocol. The fluorescent labeling pDNA/PEI_25 k_ or pDNA-GVs complexes were used to incubate with HEK293 cells or C6 cells for 6 h. After that, the cells were washed with PBS, and stained with LysoTracker Green (Thermo Fisher) for lysosome and DAPI (Sigma-Aldrich) for nuclei. Finally, the cells were observed under a confocal microscope (A1, Nikon, Japan).

### Acoustically triggering of nuclear gene delivery via intracellular cavitation of pDNA-GVs

After incubating HEK293 or C6 cells with pDNA-GVs complexes for 6 h, the free pDNA-GVs were washed out with three PBS rinses. The pDNA-GVs@HEK293 or pDNA-GVs@C6 cells were then subjected to acoustic irradiation. The following were the ultrasound parameters: The transmit frequency is 1 MHz, the acoustic pressure is 0.5 MPa, the duration is 1 min, and the duty cycle is 20%. In order to examine the location of DNA after irradiation, Cy3-labeled DNA was used, and irradiated pDNA-GVs@HEK293 or pDNA-GVs@C6 cells were stained with DAPI before being observed under a confocal microscope (A1, Nikon, Japan). A TEM was used to examine the structure of the nuclear membrane of these irradiated cells (HT7700, HITACHI, England). Morphology was examined using cytoskeleton staining. In brief, the cells were rinsed, fixed with 4% paraformaldehyde, and permeabilized with 0.1% Triton X-100 before staining with FITC-phalloidin (Sigma-Aldrich) for F-actin and DAPI (Sigma-Aldrich) for nuclei. These cells were examined under a confocal microscope after being rinsed with PBS (A1, Nikon, Japan).

### Intracellular cavitation-mediated gene transfection efficiency in vitro

The pCMV- IRES-EGFP (pEGFP) plasmid was used to create the pEGFP-GVs complexes, which were then incubated with HEK293 cells for 6 h (pEGFP-GVs@HEK293 cells). The resulting pEGFP-GVs@HEK293 cells were then subjected to 1 min of acoustic irradiation at 0, 0.1, 0.2, or 0.5 MPa pressure, followed by observation under a fluorescence microscope (EVOS M7000, Thermo Fisher, USA) after 48 h or quantitative analysis of their transfection efficiencies by flow cytometry (CytoFLEX LX, Beckman, USA). To compare the gene transfection efficiencies mediated by intracellular cavitation and extracellular cavitation, pEGFP-GVs complexes were incubated with HEK293 cells, B1610 cells, MC38 cells, and C6 cells for 6 h, yielding pEGFP-GVs@HEK293 cells, pEGFP-GVs@B1610 cells, pEGFP-GVs@MC38 cells and pEGFP-GVs@C6 cells, respectively. For 1 min, these pEGFP-GVs@cells were irradiated at 0.5 MPa of acoustic pressure. In terms of extracellular cavitation, equivalent pEGFP-GVs complexes were added to these HEK HEK293 cells, B1610 cells, MC38 cells, and C6 cells and immediately treated with acoustic irradiation at 0.5 MPa for 1 min. These irradiated cells were examined under a fluorescence microscope (EVOS M7000, Thermo Fisher, USA) after 48 h and quantitatively analyzed using flow cytometry (CytoFLEX LX, Beckman, USA).

### In vitro ultrasound imaging of pDNA-GVs@HEK293 cells

For 6 h, HEK293 cells were incubated with pEGFP-GVs. Following that, the pEGFP-GVs@HEK293 cells were rinsed with PBS and supplemented with fresh medium. After another 0 h (t_0_), 12 h (t_12_), or 24 h (t_24_) of cultivation, these pEGFP-GVs@HEK293 cells were trypsin digested and transferred into the agarose phantom wells. These cells were imaged using a high-frequency ultrasound imaging system (Vevo 2100, Visual Sonics, Canada) equipped with an MS 250 probe. The ultrasound images were obtained in contrast mode before and after these cells were given a short burst pulse to collapse the GVs in these cells. The contrast signal intensities were quantitatively analyzed using the Vevo 2100 imaging platform’s VevoCQ 1.3.12.0 analysis software.

### Time-regulated intracellular cavitation-mediated nuclear gene delivery

The EGFP plasmid was used to construct the pEGFP-GVs complexes, which were then incubated with HEK293 or C6 cells for 6 h. These pEGFP-GVs@HEK293 or pEGFP-GVs@C6 cells were rinsed with PBS to remove free pEGFP-GVs complexes before being supplemented with a fresh medium. After another 0 h (t_0_), 12 h (t_12_), or 24 h (t_24_) of cultivation, the pEGFP-GVs@HEK293 or pEGFP-GVs@C6 cells were exposed to the acoustic irradiation at 0.5 MPa for 1 min. After 48 h, the expression of EGFP in irradiated and non-irradiated cells was examined using a fluorescence microscope (EVOS M7000, Thermo Fisher, USA) and quantified using flow cytometry (CytoFLEX LX, Beckman, USA).

### In vitro wound healing assay

The pReceiver-M83 expression plasmid with the *E-cadherin* gene insertion (NM_009864.2) was purchased from GeneCopoeiaTM. This recombinant plasmid (named pCMV-E-cadherin-IRES-mCherry, pEcad) contains an internal ribosome entry site (IRES) that allows the mCherry fluorescent protein to be expressed simultaneously. The negative control was the same plasmid without the E-cadherin insertion (named pCMV-IRES-mCherry, pCherry). The resulting pEcad or pCherry control plasmid was used to generate the pCherry-GVs or pEcad-GVs complexes, which were then incubated with C6 cells for 6 h. After a PBS rinse, pCherry-GVs@C6 cells or pEcad-GVs@C6 cells were cultured for another 0 h (t_0_), 12 h (t_12_), or 24 h (t_24_), with or without 0.5 MPa acoustic irradiation for 1 min. The free cells were then removed by drawing a vertical line from the upper edge of each well and adding PBS. A fresh medium was used to incubate the cells. Microscopy was used to examine the wound healing and cell morphology after 48 h (EVOS M7000, Thermo Fisher, USA). Image J software was used to calculate the wound healing area.

### In vitro tumor cell migration and invasion assay

The C6 cells were incubated with pCherry-GVs or pEcad-GVs complexes for 6 h. After a PBS rinse, pCherry-GVs@C6 cells or pEcad-GVs@C6 cells were cultured for another 0 h (t_0_), 12 h (t_12_), or 24 h (t_24_), with or without 0.5 MPa acoustic irradiation for 1 min. Following that, trypsin digestion was used to collect these pCherry-GVs@C6 cells or pEcad-GVs@C6 cells. The cells were then transferred in 200 µL of serum-free DMEM medium at a density of 1.5 × 10^5^ per well in uncoated or Matrigel-coated (BD Biosciences) Transwell inserts (pore size 8 µm). The lower chambers were filled with 600 µL of DMEM supplemented with 10% FBS. After 48 h, the non-migrated cells in the upper chamber were removed, and those on the lower surface of the insert were fixed with 70% anhydrous ethanol for 10 min before being stained with a diluted Giemsa solution (Yeasen) for 15–30 min. The stained cells were photographed and quantified in four non-overlapping random fields using a microscope (EVOS M7000, Thermo Fisher, USA).

### Real-time quantitative PCR assay

The RNA was extracted from wild-type C6 cells and irradiated or non-irradiated pDNA-GVs@C6 cells using TRIzol (Invitrogen) reagent, following the manufacturer’s instructions. The strand of RNA was then reverse-transcribed to complementary DNA. The resulting cDNA was amplified and analyzed using a CFX ConnectTM Real-Time PCR System and an SYBR® Green PCR Supermix kit (Vazyme) (Bio-Rad, Hercules, USA). The expression of each gene was normalized against that of glyceraldehyde-3-phosphate dehydrogenase (GAPDH) expression. The 2^−∆∆Ct^ method was used to quantify relative gene expression in qPCR. The primer sequences used for qPCR are detailed in Supplementary Table 1.

### In vivo tumor invasion inhibition of the acoustically triggering nuclear *E-cadherin* delivery in the orthotopic tumor mouse model

Nude mice were obtained from Beijing Vital River Laboratory Animal Technology Co. Ltd., and all animal experiments were carried out in accordance with protocols approved by the Ethics Committee of Shenzhen Institutes of Advanced Technology, Chinese Academy of Sciences. Nude mice were used to create an orthotopic glioma mouse model (6–8 weeks of age). In brief, 5 × 10^5^ pEcad-GVs@C6 cells in 5 µL PBS were intracranially injected into the right hemisphere of the murine brains (2 mm right lateral to bregma and 3 mm depth) using a stereotactic fixation device. After waiting for 0 h (t_0_), 12 h (t_12_) or 24 h (t_24_), these transplanted pEcad-GVs@C6 cells were treated with or without acoustic irradiation at 0.5 MPa for 1 min. The survival time of tumor-bearing mice was recorded (nine mice in each group). Three mice per group were sacrificed at 21 days for histological analysis of tumor invasion to evaluate the tumor invasion inhibition effects of intracellular cavitation-mediated nuclear *E-cadherin* delivery in vivo. Hematoxylin and Eosin (H&E) staining was performed on whole brain sections from each mouse across the tumor. Sections were also immunofluorescently stained to assess tumor cell proliferation potential. Anti-PCNA (Abcam) and anti-Ki67 (Abcam) antibodies were used. The tumor cell invasion distance was calculated by measuring the distance from the tumor core to the furthest tumor spread distance. The invasion volume of tumor cells was calculated by multiplying the invasion area by the invasion distance.

### In vivo tumor migration inhibition of the acoustically triggering nuclear *E-cadherin* delivery in the subcutaneously transplanted tumor model

First, 1 × 10^6^ pEcad-GVs@C6-Luc cells were subcutaneously injected into the right back of neonatal Wistar rats (within 24 h of birth) in 40 µL PBS.^50^ These transplanted pEcad-GVs@C6-Luc cells were treated with or without acoustic irradiation at 0.5 MPa for 1 min after waiting for 0 h (t_0_), 12 h (t_12_), or 24 h (t_24_). The tumor-bearing mice’s survival time was recorded. Three rats from the t_0_ group were sacrificed on the fifth day after transplantation to assess in vivo mCherry expression. The tumors were excised and imaged using the IVIS small animal imaging system (IVIS® Spectrum, PerkinElmer, USA) at 580 ± 20 nm excitation and 610 ± 30 nm emission at 580 ± 20 nm excitation. Three rats per group were sacrificed on the 35th day after transplantation to assess the tumor migration inhibition effects of nuclear *E-cadherin* delivery in vivo. These rats were given an intraperitoneal injection of D-Luciferin potassium salt before being sacrificed (PerkinElmer). The lungs of rats were excised and imaged using the IVIS system, and then, the lung weight was recorded. The histological analysis was then carried out to assess the metastatic nodules in the lung. Other rats (*n* = 8 in each group) underwent survival analysis using the Kaplan-Meier method.

### Nuclear gene delivery at different cell cycle phases

To determine the gene expression efficiency of the acoustically triggering nuclear gene delivery at different phases of the cell cycle, C6 cells were first synchronized in the G1 phase with 20 µM lovastatin (Sigma-Aldrich) for 24 h, S phase with 5 mM double-thymidine block treatment (Sigma-Aldrich) with 18 h for second cell-cycle block or G2/M phase with 100 nM nocodazole (Sigma-Aldrich) for 18 h. The ratios of cells successfully synchronized in given phases were confirmed by flow cytometry (CytoFLEX LX, Beckman, USA). For 6 h, the pEcad-GVs complexes were incubated with C6 cells synchronized in different cell cycle phases. After a PBS rinse, the synchronized pEcad-GVs@C6 cells were treated with or without acoustic irradiation at 0.5 MPa for 1 min and cultured for another 48 h before being analyzed by flow cytometry for E-cadherin expression efficiency (CytoFLEX LX, Beckman, USA). C6 cells were synchronized in the G2/M phase with 100 nM nocodazole for 12 h before pEcad-GVs were added to the medium for uptake by these cells for 6 h to determine the expression efficiency of nuclear gene delivery in a running cell cycle. The cells were then washed with PBS to remove the nocodazole. The G2/M-synchronized pEcad-GVs@C6 cells were released in a fresh nocodazole-free medium for 0 h (G2/M phase), 8 h (G1 phase), or 14 h (S phase), and then treated with or without acoustic irradiation at 0.5 MPa for 1 min. These irradiated cells and their non-irradiated counterparts were used to analyze the expression efficiency of E-cadherin by flow cytometry 48 h after acoustic irradiation (CytoFLEX LX, Beckman, USA). The control cells were asynchronized pCherry-GVs@C6 cells. Furthermore, the C6 cells were synchronized in the G1 phase for 18 h with 20 µM lovastatin before being incubated with pEcad-GVs for 6 h. The G1-synchronized pEcad-GVs@C6 cells were released in a fresh lovastatin-free medium for 0 h (G1 phase), 6 h (S phase) or 8 h (G2/M phase), and then treated as described above. As for the Transwell invasion assay of C6 cells received with nuclear *E-cadherin* gene delivery at different cell cycle phases. The synchronized pEcad-GVs@ C6 cells in the G2/ M phase were cultured in a fresh medium for 0 h (G2/M phase), 8 h (G1 phase) or 14 h (S phase) and then treated with or without acoustic irradiation at 0.5 MPa for 1 min. Similarly, the synchronized pEcad-GVs@C6 cells in the G1phase were cultured in a fresh medium for 0 h (G1 phase), 6 h (S phase) or 8 h (G2/M phase) and then treated with or without acoustic irradiation at 0.5 MPa for 1 min. After irradiation, the irradiated cells and their non-irradiated counterparts were transferred into the Transwell inserts, and the migrated cells were stained with a diluted Giemsa solution for 15–30 min before being examined and quantified in four non-overlapping random fields under a microscope (EVOS M7000, Thermo Fisher, USA).

### In vivo tumor migration inhibition of the acoustically triggering nuclear *E-cadherin* delivery in different phases

First, 1 × 10^6^ synchronized G2/M pEcad-GVs@C6-Luc cells were resuspended in 40 µL PBS and subcutaneously injected into the right back of neonatal Wistar rats. After 0 h (G2/M phase), 8 h (G1 phase), or 14 h (S phase), these rats were transplanted with pEcad-GVs@C6-Luc cells and treated with or without 0.5 MPa acoustic irradiation for 1 min. To confirm mCherry expression, tumors from three rats treated with or without acoustic irradiation were removed. The tumor-bearing mice’s survival time was recorded. Similar previous methods were used to examine the lung tissue histologically.

### RNA-sequencing analysis

In the G2/M phase, synchronized pCherry-GVs@C6 cells or pEcad-GVs@C6 cells were exposed to 0.5 MPa acoustic irradiation for 1 min. After 48 h, mCherry-positive cells from irradiated pCherry-GVs@C6 or pEcad-GVs@C6 cells were sorted using fluorescence-activated cell sorting (FACSAria™ III, BD, USA). RNA was extracted from cells using the TRIzol (Invitrogen) reagent according to the manufacturer’s instructions. To build sequencing libraries, total RNA (1 µg per sample) was used. mRNA was enriched and cleaved into short fragments using a fragmentation buffer, then reverse transcribed into cDNA using random primers. Purified cDNA fragments were end-repaired, and a poly(A) tail was added before ligation with Illumina sequencing adapters. The ligation products were then size-sorted using agarose gel electrophoresis, amplified using PCR, and sequenced on an Illumina Novaseq 6000. (Gene Denovo Biotechnology). Differentially expressed genes (DEGs) were identified using differential RNA expression analysis. Transcripts with a False Discovery Rate (FDR) of less than 0.05 and an absolute fold change of 1.5 or greater were classified as differentially expressed. The Kyoto Encyclopedia of Genes and Genomes (KEGG) and Gene Ontology were used to perform pathway enrichment analysis (GO). The calculated p-value was FDR corrected, with a threshold of 0.05 or less. This condition was met by pathways that were significantly enriched in differentially expressed genes.

### qPCR and western blotting analysis of the relationship between E-cadherin and Fam50a

qPCR and western blotting were used to determine the mRNA and protein levels of Fam50a in E-cadherin up-regulated or down-regulated C6 cells in the G2/M phase. C6 cells were synchronized in the G2/M phase for 18 h with 100 nM nocodazole before being transfected with pEcad plasmid or siE-cadherin using GP-transfect-Mate (GenePharma) according to the manufacturer’s protocol. Total RNA was extracted from cells using the TRIzol (Invitrogen) reagent, and mRNA levels of E-cadherin and Fam50a were measured using qPCR, as previously described. Furthermore, the protein levels of E-cadherin and Fam50a were measured in these cells using anti-E-cadherin (Abcam) and anti-Fam50a antibodies (Abcam). Transwell experiments also confirmed the functional relationship between E-cadherin and Fam50a. In brief, G2/M-phase synchronized C6 cells were transfected with pEcad plasmid or a 1:1 mixture of pEcad plasmid/pCMV-Fam50a-IRES-mCherry plasmid via acoustic nuclear gene delivery, or with siE-cadherin or siE-cadherin/siFam50a using GP-transfect-Mate (GenePharma). These transfected cells were transferred into the Transwell inserts 48 h after acoustic irradiation or siRNA transfection, and their invasion potential was evaluated using the methods described above. After 48 h, the protein levels of E-cadherin and Fam50a in these transfected cells were also measured. The sequences used for siRNA are presented in Supplementary Table 2.

### Co-IP analysis of the relationship between Fam50a and Runx2

Through acoustic triggering of nuclear gene delivery, synchronized C6 cells in the G2/M phase were transfected with pECMV-Runx2-m-FLAG plasmids or pECMV-Fam50a-m-FLAG/pECMV-Runx2-m-FLAG (1:1) plasmids. The transfected cells were harvested and lysed with RIPA solution. Then, anti-IgG beads (Sigma-Aldrich) were used for immunoprecipitation along with antibody-Fam50a or antibody-Runx2, and the precipitate was washed four times using cell lysis buffer, and the proteins were resolved on a western blot.

### Promoter transcription activity assay

The MMP13 and SPP1 promoter sequences were inserted into the pCMV-C-Luciferase vector to create the plasmids pMMP13-C-Luc and pSPP1-C-Luc, respectively. MMP13 promoter transcription activity was examined in Runx2-overexpressed/Fam50a-overexpressed cells and Runx2-overexpressed/Ecad-overexpressed cells. In brief, G2/M-synchronized C6 cells were transfected with plasmids pMMP13-C-Luc/pECMV-Runx2-m-FLAG, pMMP13-C-Luc/pECMV-Fam50a-m-FLAG/pECMV-Runx2-m-FLAG (at 1:1:1 or 1:2:1 ratio), or pMMP13-C-Luc/pCMV-E-cadherin (at 1:1:1 or 1:2:1 ratio). D-Luciferin potassium salt (PerkinElmer) was added to the transfected cells 48 h later, and the fluorescent intensity was measured using a microplate reader (Infinite 200 pro, TECAN, Switzerland). MMP13 promoter transcription activity was also examined in Runx2-overexpressed/Fam50a-knockdown and Runx2-overexpressed/Ecad-knockdown cells. Briefly, G2/M-synchronized C6 cells were transfected with the plasmids pMMP13-C-Luc/pECMV-Runx2-m-FLAG. These cells were also transfected with various doses of siFam50a or siE-cadherin using GP-transfect-Mate (GenePharma) according to the manufacturer’s instructions. Furthermore, the promoter transcription activity of MMP13 was investigated in Runx2-deficient/Fam50a-overexpressed or Runx2-deficient/E-cad-overexpressed cells. These cells were transfected with siRunx2, and either plasmids pMMP13-C-Luc/pECMV-Fam50a-m-FLAG or pMMP13-C-Luc/pCMV-E-cadherin-IRES-mCherry (1:1 or 1:2 ratio). As previously stated, the fluorescent intensity was measured 48 h after transfection. Similarly, the promoter transcription activity of SPP1 was investigated by replacing the pMMP13-C-Luc plasmid with the pSPP1-C-Luc plasmid.

### Transcription levels of Fam50a/Runx2-MMP13 in different cell cycle phases in the E-cadherin overexpressed cells

Acoustically triggered nuclear gene delivery was used to transfect synchronized C6 cells in the G1, S, or G2/M phases with the pEcad plasmid. The control cells were non-irradiated pEcad-GVs@C6 cells. The total mRNA from these transfected cells was isolated, and the transcription levels of the E-cadherin, Fam50a, Runx2, MMP13, and SPP1 genes were determined using qRCR analysis, as previously described.

### MMP13 reduces the tumor cell invasion inhibition effects from overexpression of E-cadherin

The open reading frame of the MMP13 gene (NM_133530.1) was cloned into the pCMV-C-EGFP vector to develop the pCMV-MMP13-C-EGFP plasmid. Acoustically triggered nuclear gene delivery was used to transfect synchronized C6 cells in the G2/M phase with pCherry, pEcad, or pEcad/pCMV-MMP13-C-EGFP plasmids. After acoustic irradiation, the cells were transferred into Transwell inserts precoated with Matrigel, and the migrated cells were stained with a diluted Giemsa solution for 15–30 min before being examined and quantified in four non-overlapping random fields under a microscope (EVOS M7000, Thermo Fisher, USA).

### Statistical analysis

GraphPad Prism 9.0 was used for statistical analysis. All data are presented as the mean ±standard deviation. When comparing two or more groups, the unpaired Student’s t-test or one-way analysis of variance (ANOVA) were used. For the comparison of survival curves, a log-rank test was used; *P* < 0.05 was considered significant.

## Supplementary information

Supplementary

## Data Availability

All RNA-seq raw data of this study have been deposited in BIG Data Center (Submission ID: PRJCA014812). The other resource data supporting this paper are presented within the Supplementary Materials. The data that support the findings of this study are available from the authors upon reasonable request.
