# Supplementary material for: Cell-cycle dependent nuclear gene delivery enhances the effects of E-cadherin against tumor invasion and metastasis
Source: Signal Transduct Target Ther. 2023 May 8;8:182. doi: 10.1038/s41392-023-01398-4 (PMC10164743; doi:10.1038/s41392-023-01398-4)
Supplement: Supplementary file 1 — Supplementary [file 41392_2023_1398_MOESM1_ESM.doc]

**Supplementary Materials for**

**Cell-cycle dependent nuclear gene delivery enhances the effects of E-cadherin against tumor invasion and metastasis**

Liting Xie ^1^, Jieqiong Wang ^2^, Liming Song ^3^, Tianan Jiang ^1*^, Fei Yan ^2*^

* Correspondence to: Fei Yan ([fei.yan@siat.ac.cn](mailto:fei.yan@siat.ac.cn)) and Tianan Jiang ([tiananjiang@zju.edu.cn](mailto:tiananjiang@zju.edu.cn))

**This PDF file includes:**

Supplementary Methods

Supplementary Figures. S1 to S20

Supplementary Tables S1 to S2

**Supplementary Methods**

**1.1 DNA binding capability of GVs.**

The ability of GVs to bind DNA was investigated using a gel retardation assay. For 30 min at 37 °C, the GVs were incubated with DNA/PEI at various N/P ratios (1:1, 2:1, 5:1, 10:1, or 15:1). Then, the pDNA-GVs complexes were electrophoresed on a 1% agarose gel. The gel was then imaged using a gel imaging device (Gel Doc XR+, Bio-Rad, US).

### 1.2 Cell culture.

### HEK293, B1610, MC38, and C6 cells were obtained from the Chinese Academy of Sciences' Cell Bank of Type Culture Collection (ATCC, Shanghai, China). All of these cells were cultured at 37 °C under 5% CO_2_ in Dulbecco's modified Eagle medium (DMEM) supplemented with 10% fetal bovine serum (FBS) and 1% penicillin/streptomycin.

**1.3 *In vitro* cell viability assay.**

Cell viability was measured using Cell Counting Kit-8 (CCK-8) according to the manufacturer's protocol after incubating HEK293 cells at a density of 5 × 10^4^ with GVs or pDNA-GVs complexes for 0 h, 12 h, 24 h, or 48 h. (Dojindo). The optical density (OD) was measured at 450 nm using a microplate reader (Tecan, Infinite 200 Pro NanoQuant, Austria). The cell viability was calculated using the following formula: Cell viability = mean (OD of treated cells minus OD of control cells)/mean (OD of control-OD of blank). The GVs@HEK293 cells or pDNA-GVs@HEK293 cells were obtained by incubating HEK293 cells with GVs or pDNA-GVs complexes for 6 h and then treating them with or without acoustic irradiation. The following acoustic parameters were used: transducer central frequency = 1 MHz, acoustic pressure = 0.5 MPa, duration = 1 min, duty cycle = 20%. The control cells were plain HEK293 cells that had received the same acoustic irradiation. Cell viability was determined after 24 h, as previously described.

**Supplementary Figures**

**Fig.S1.**


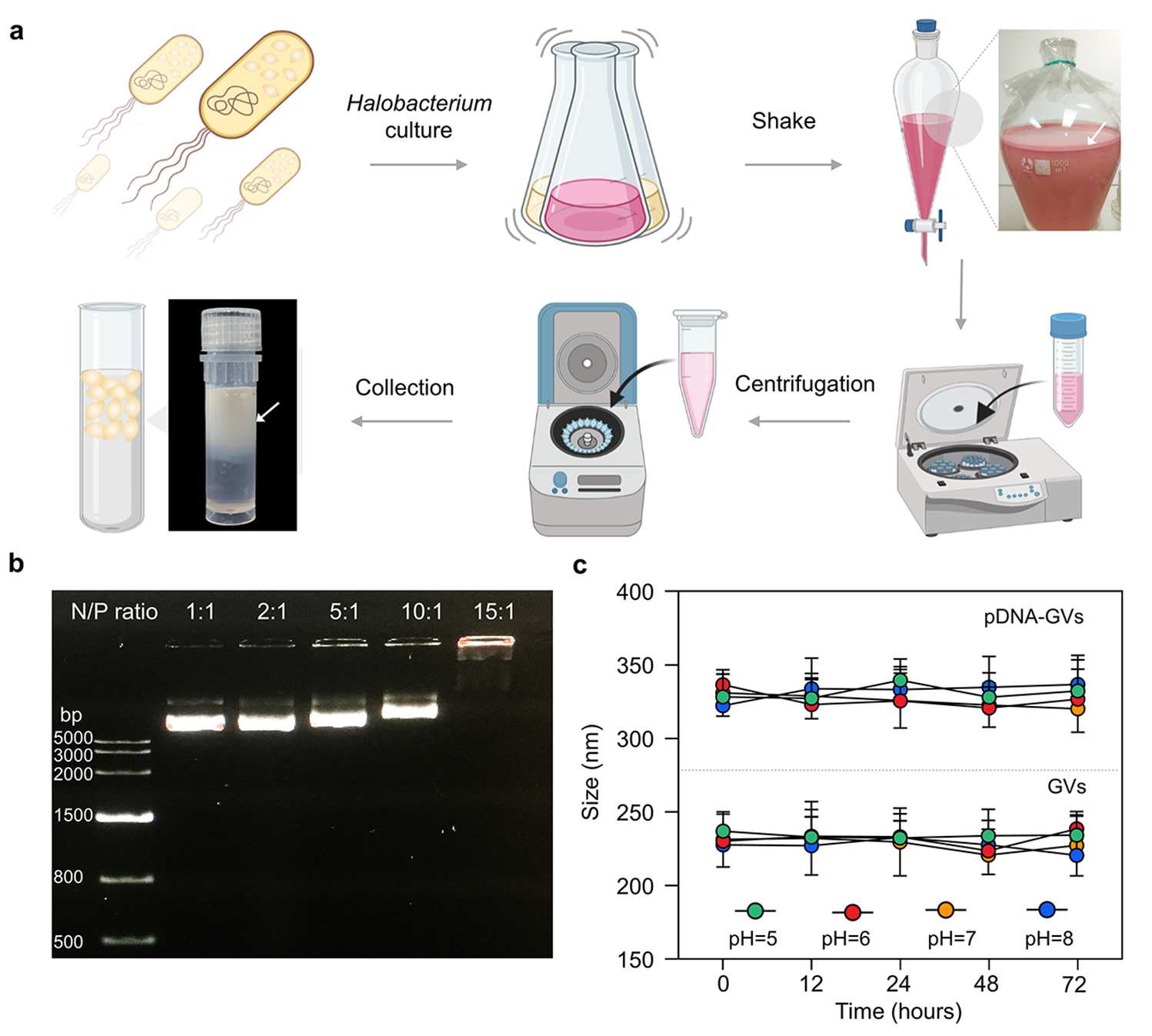


**Fig.S1.** **Synthesis, DNA-binding capability of GVs and stability of pDNA-GVs**.

**a**, Schematic illustration of the synthesis and purification procedure of GVs. Created with BioRender. **b**, Analysis of Agarose gel electrophoresis analysis for the DNA binding capability of GVs at different N/P ratios (1:1, 2:1, 5:1, 10:1, or 15:1). **c**, Stability of GVs and pDNA-GVs. Samples in PBS at different pH (pH5, 6, 7 or 8) for a given time (0, 6, 12, 18 or 24 h) were evaluated by analyzing their particle size.

**Fig.S2.**


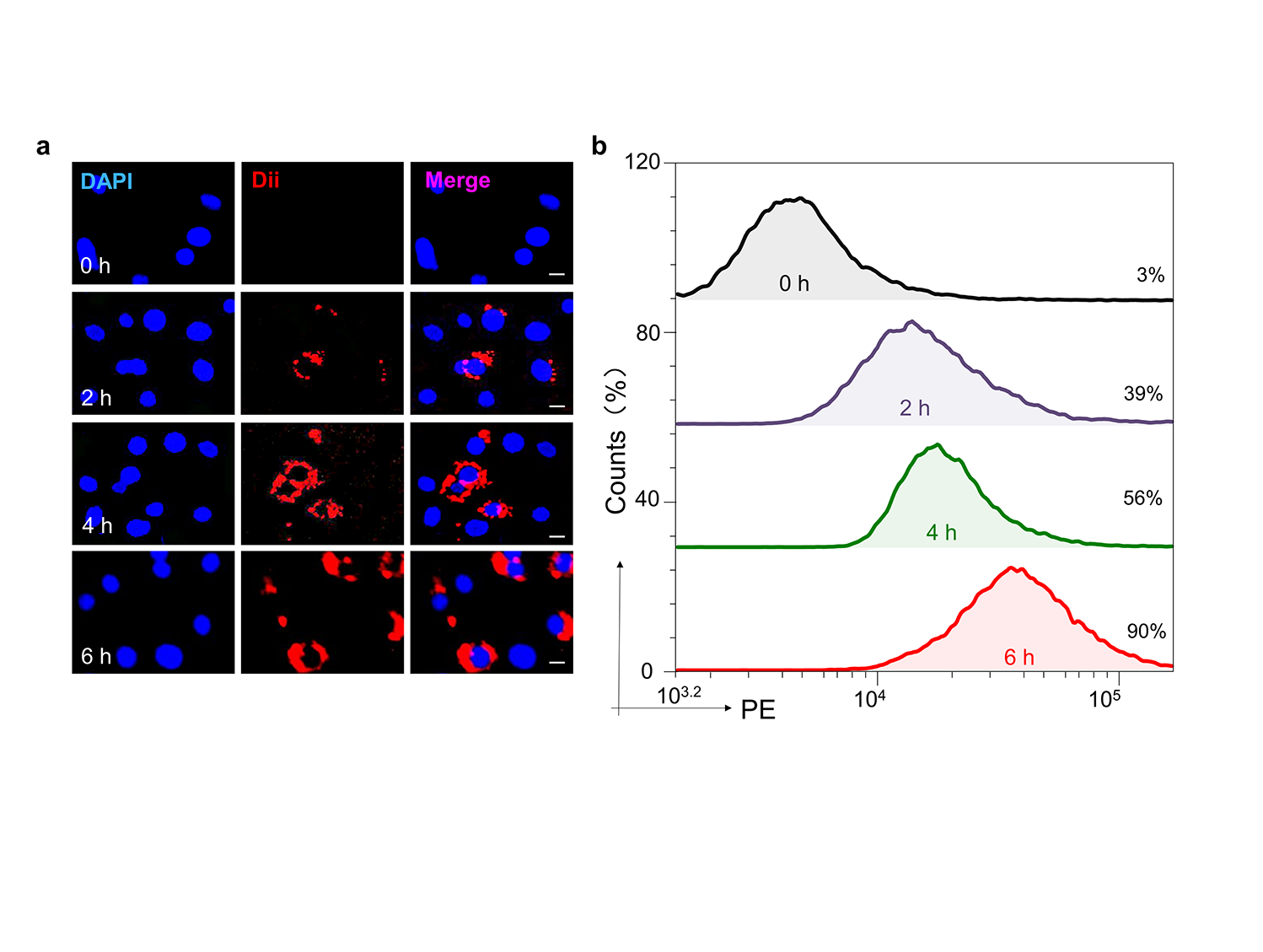


**Fig.S2.** **The cellular uptake of pDNA-GVs**.

**a**, Fluorescence microscope images of C6 cells incubated with fluorescently labeled pDNA-GVs (red) for 0, 2, 4 or 6 h. The nuclei were stained with DAPI (blue). Scale bar = 10 µm. **b**, Flow cytometry analysis of the intensities of the fluorescent signals of C6 cells from supplementary Fig.2a.

**Fig.S3.**


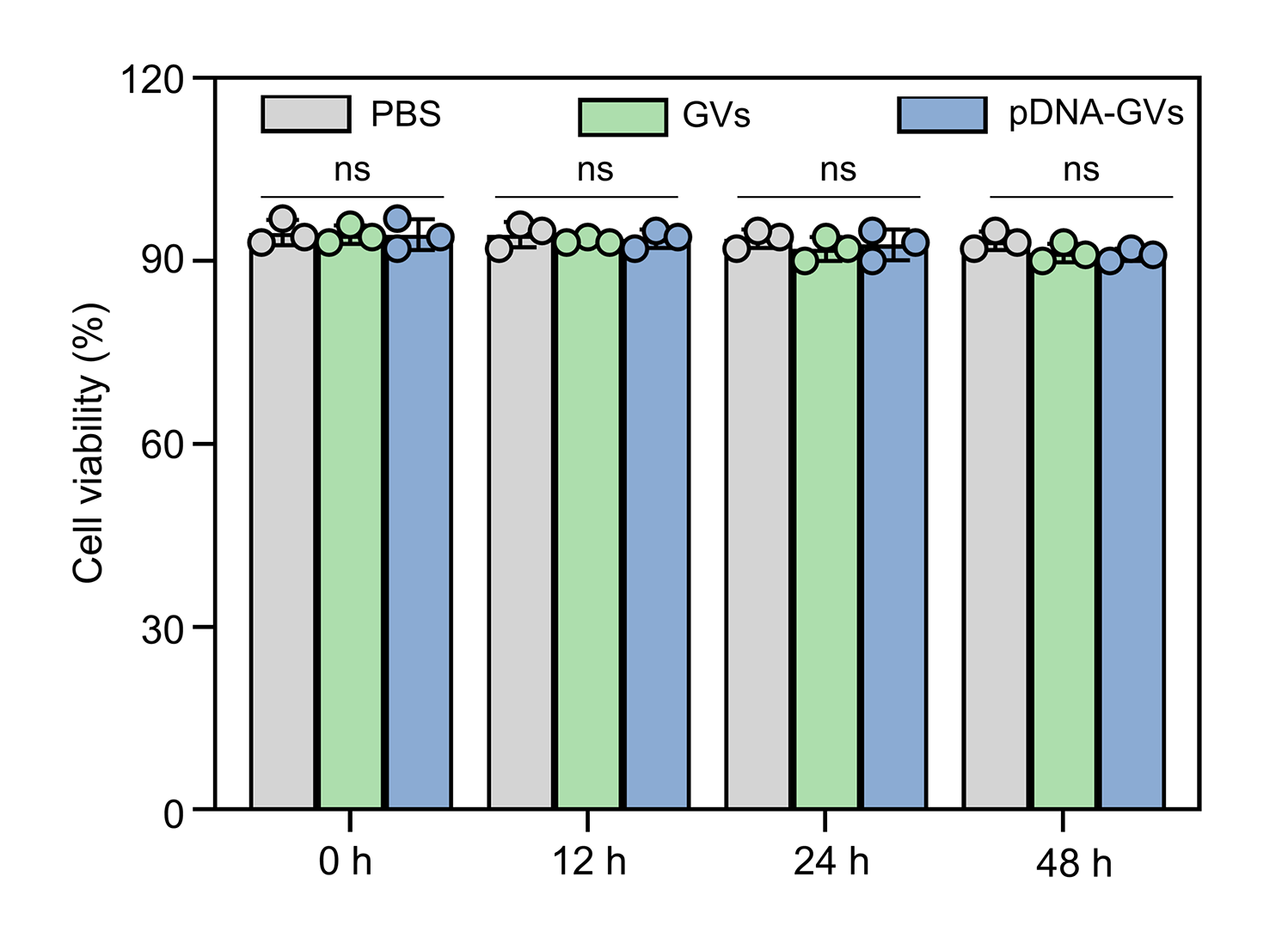


**Fig.S3.** ***In vitro* cell viability of GVs@HEK293 cells and pDNA-GVs@ HEK293 cells**.

Cell viability was measured by the CCK8 assay after incubating GVs or pDNA-GVs with HEK293 cells for 0, 12, 24, or 48 h.

**Fig.S4.**


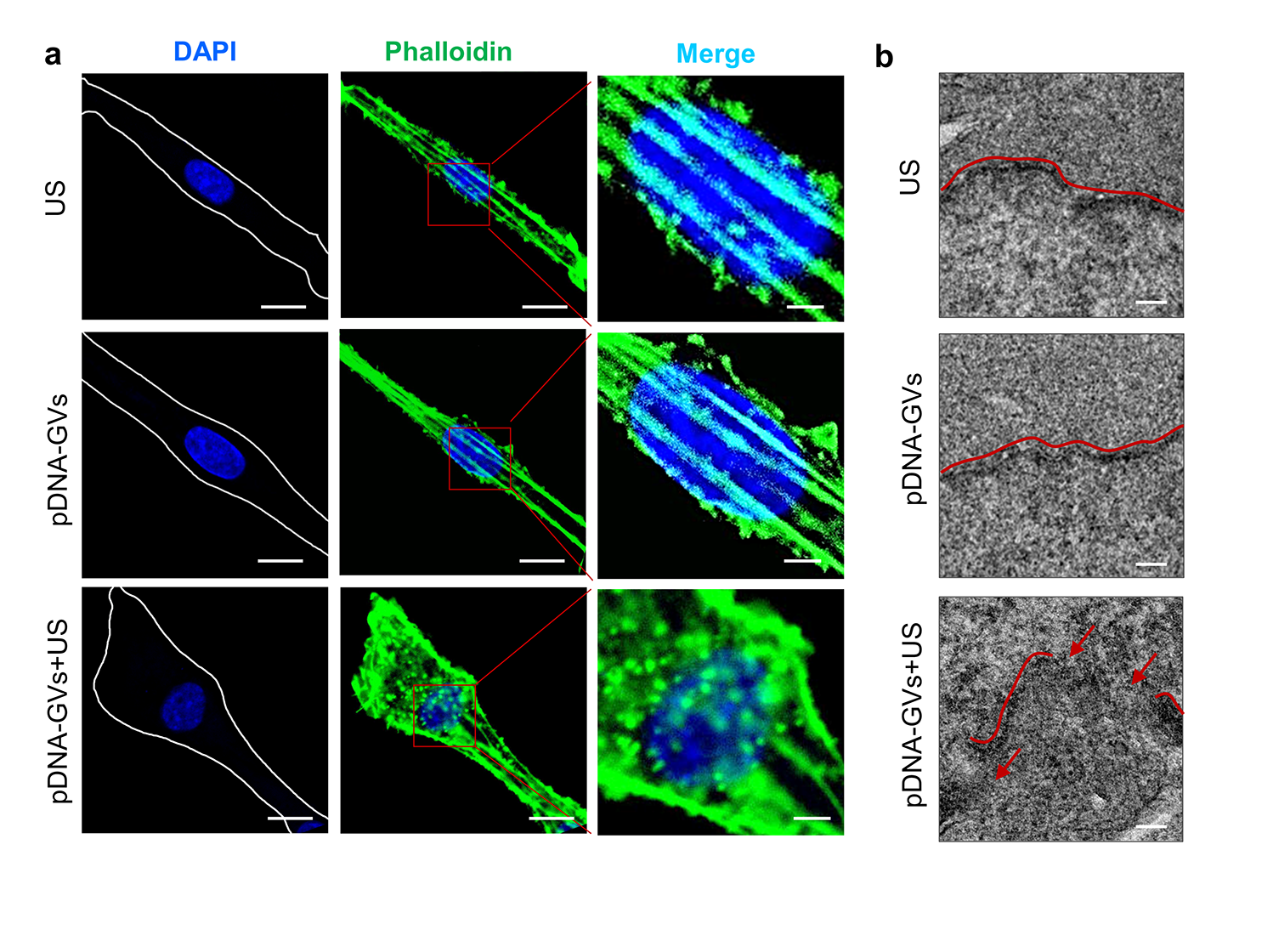


**Fig.S4.** **Cytoskeleton staining and TEM of C6 cells after intercellular cavitation.**

**a**, Fluorescent staining images of C6 cells for the cytoskeleton with FITC-phalloidin (green) and for the nuclei with DAPI (blue), showing the messy orientation of the cytoskeleton after intracellular cavitation. Scale bar = 5 µm (left and middle panels), Scale bar = 2 µm (right panel). **b**, TEM images of C6 cells after intracellular cavitation, showing the destructed nuclear membrane (Red arrows). The nuclear membrane was marked by red lines. Scale bar = 200 nm.

**Fig.S5.**


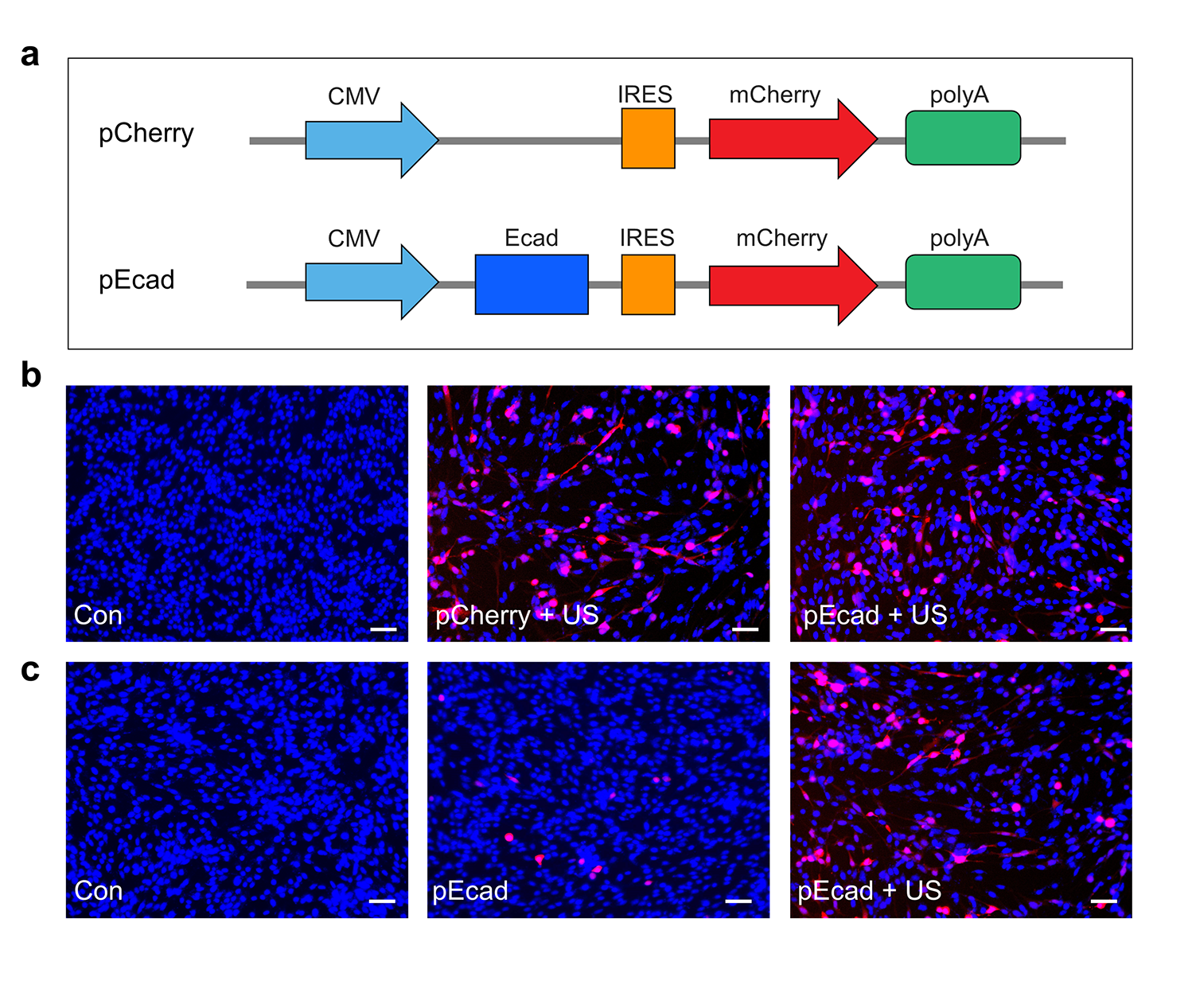


**Fig.S5.** **Nuclear delivery of the** **E-cadherin gene via intracellular cavitation.**

**a** Schematic illustration of the vector pCMV-IRES-mCherry (pCherry) or pCMV-E-cadherin-IRES-mCherry (pEcad). **b,** Representative fluorescence imaging of pCherry-GVs@C6 or pEcad-GVs@C6 cells 24 h after acoustic irradiation. Plain C6 cells that did not receive acoustic irradiation were used as control. Scale bar = 100 µm. **c**, Representative fluorescence imaging of pEcad-GVs@C6 cells received with or without acoustic irradiation. Scale bar = 100 µm.

**Fig.S6.**


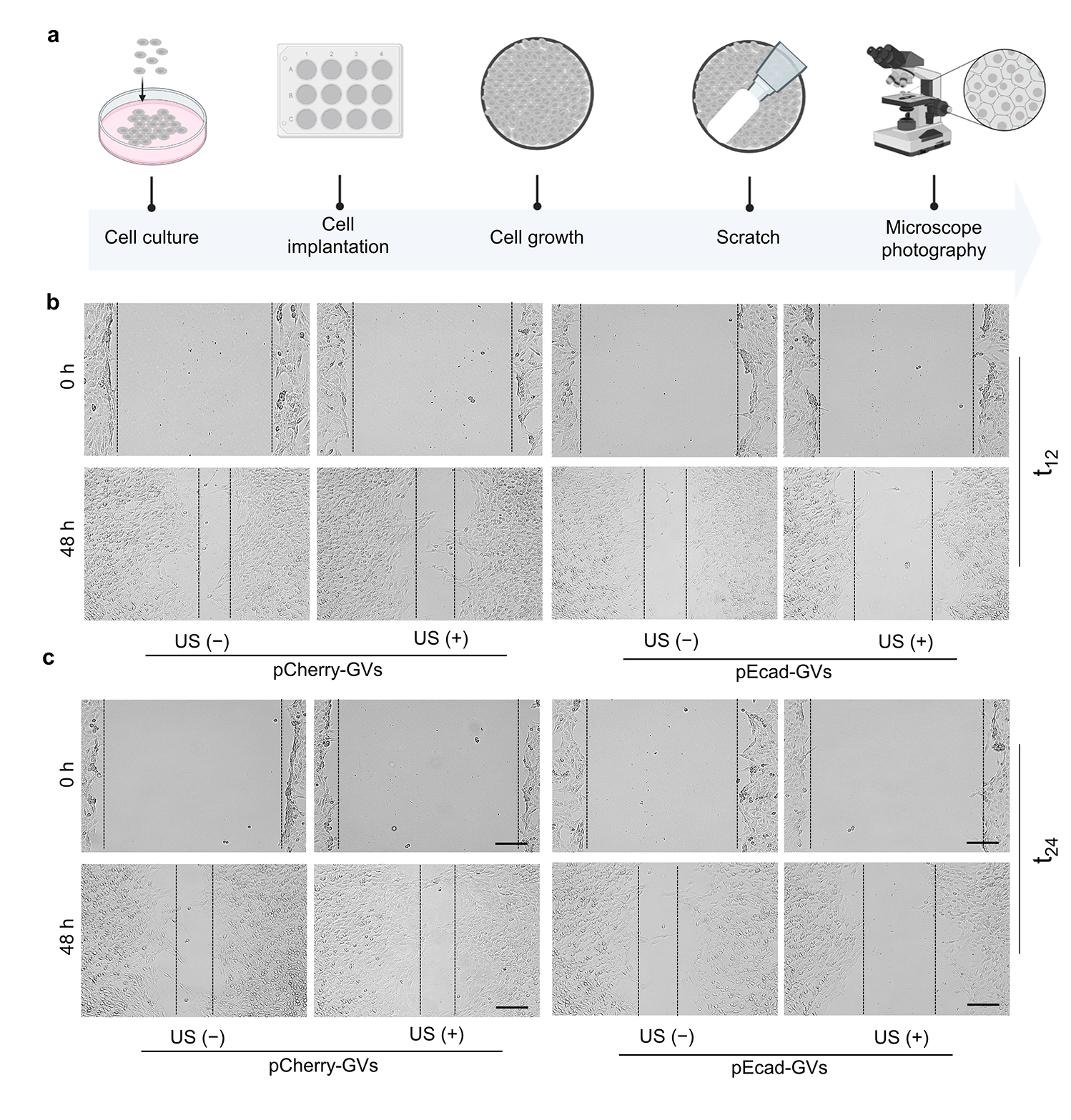


**Fig.S6.** **Temporal control of nuclear *E-cadherin* delivery inhibits tumor cell migration**

**a,** Schematic illustration of the wound healing assay. Created with BioRender. **b, c,** Representative bright images of the wound healing assay for pCherry-GVs@C6 cells or pEcad-GVs@C6 cells that received with or without nuclear gene delivery at t_12_ and t_24_, respectively. Scale bar = 100 µm.

**Fig.S7.**


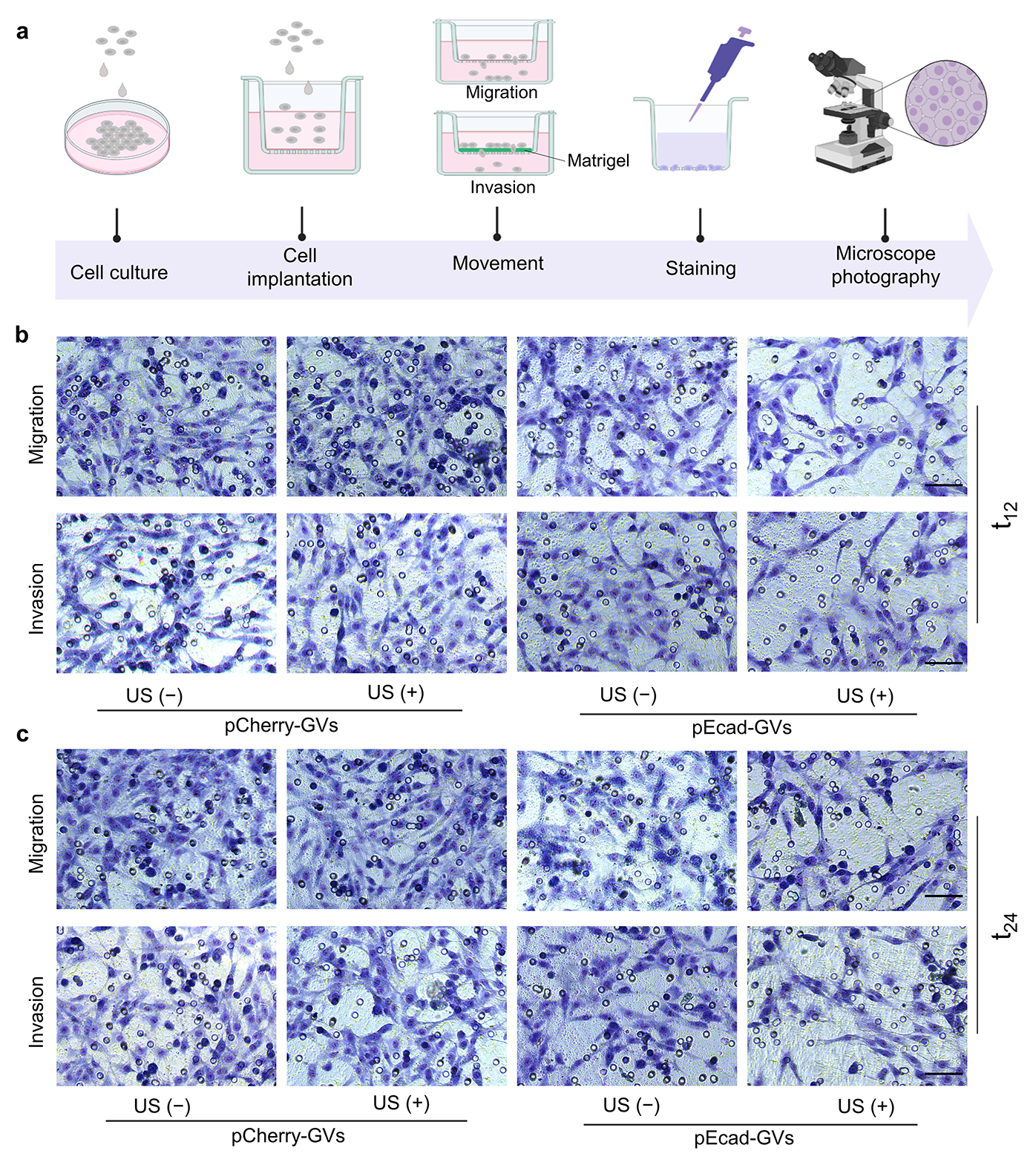


**Fig.S7.** **Temporal control of nuclear *E-cadherin* delivery inhibits tumor cell migration and invasion**

**a,** Schematic illustration of the Transwell assay. Created with BioRender. **b,c,** Representative microscope images of migrated or invaded pCherry-GVs@C6 cells or pEcad-GVs@C6 cells treated with or without acoustic irradiation at t_12_ and t_24_, respectively. Scale bar = 25 µm.

**Fig.S8.**


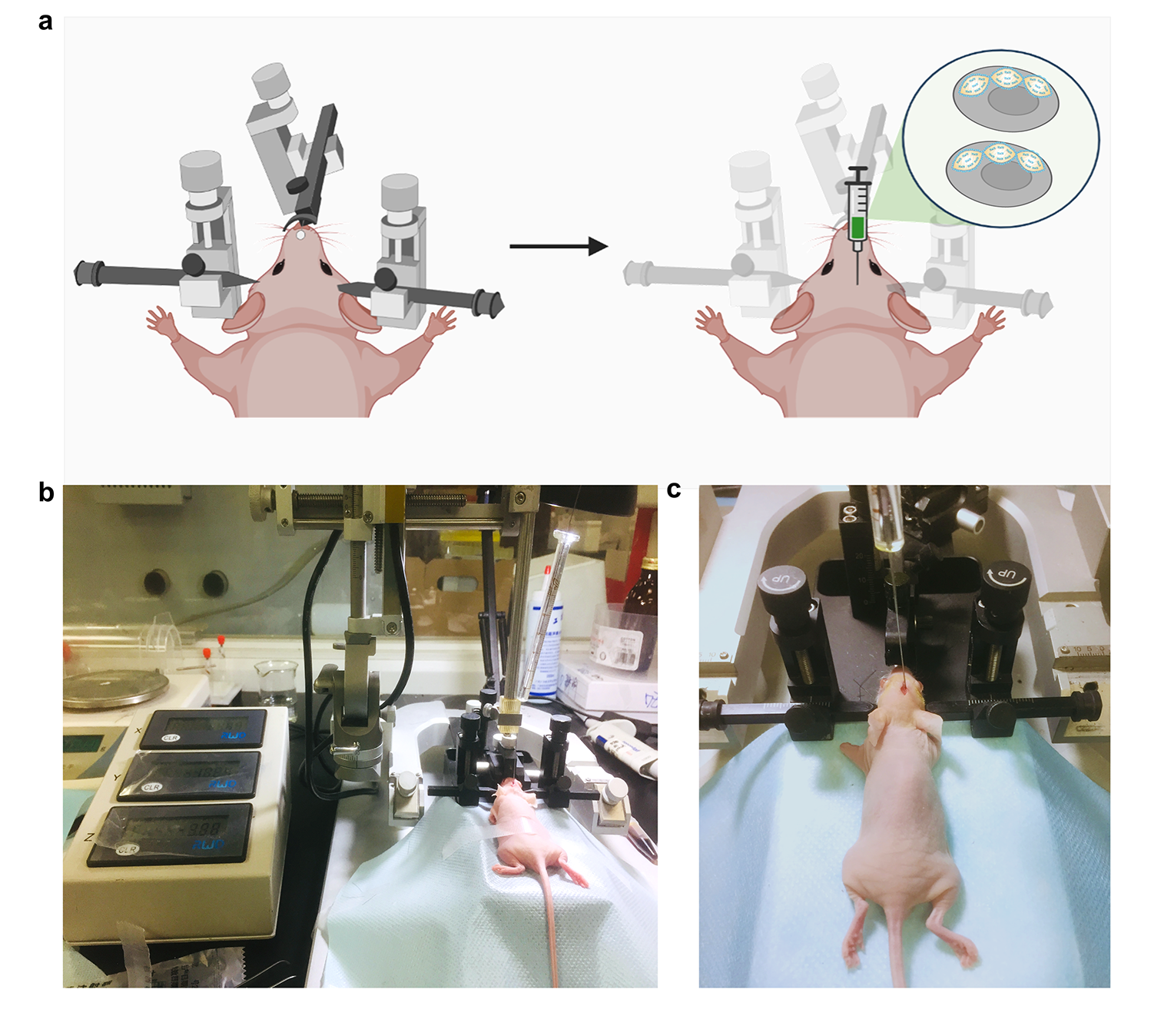


**Fig.S8. *In vivo* orthotopic glioma model.**

**a**, Schematic illustration of the establishment of an orthotopic glioma model. A stereotaxic apparatus was used to aid in the injection of tumor cells. Created with BioRender. **b,c**, Representative images of the model establishment when pEcad-GVs@C6 cells were orthotopically transplanted into the brain of mice.

**Fig.S9.**


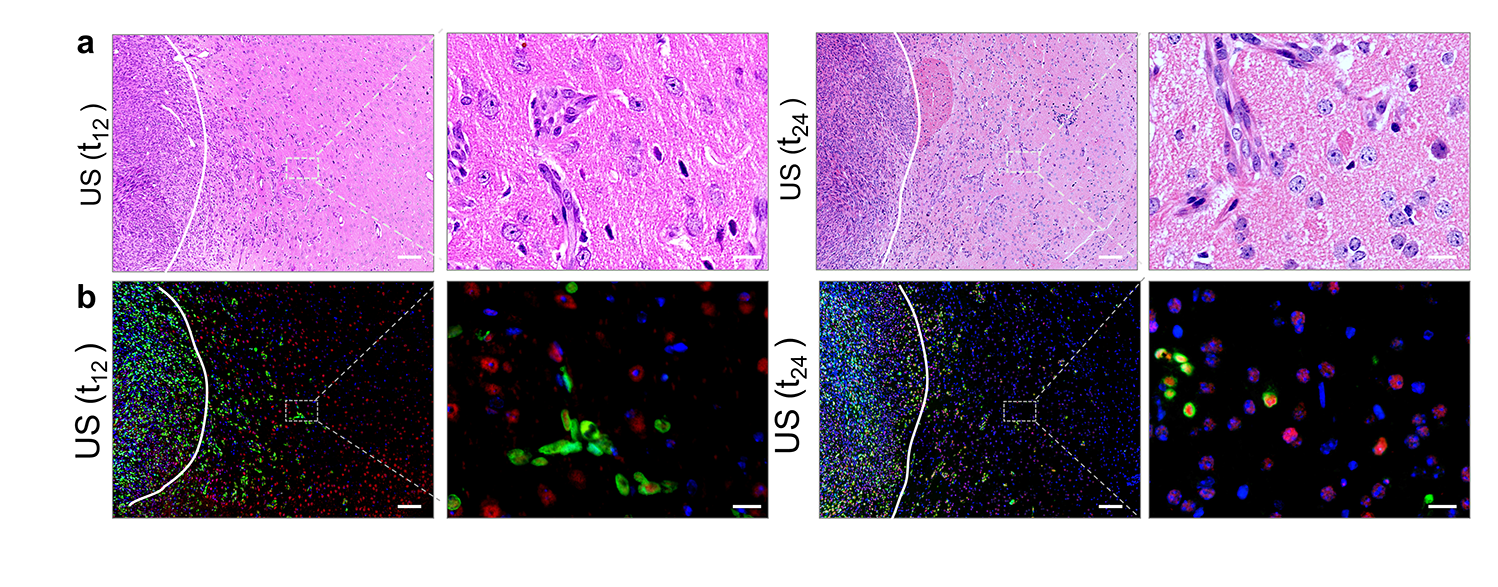


**Fig.S9. Intracellular cavitation-mediated E-cadherin delivery inhibited tumor invasion *in vivo*.**

Representative immunofluorescence staining images of brain tumor sections stained with DAPI (blue), anti-PCNA antibody (red), and anti-Ki67 antibody (green) for the US(-) and US(t_0_) groups. Scale bar = 100 µm. The local enlarged images were from the peritumoral regions with equal distances from the boundaries of the main tumor mass (right panel). Scale bar = 20 µm.

**Fig.S10.**


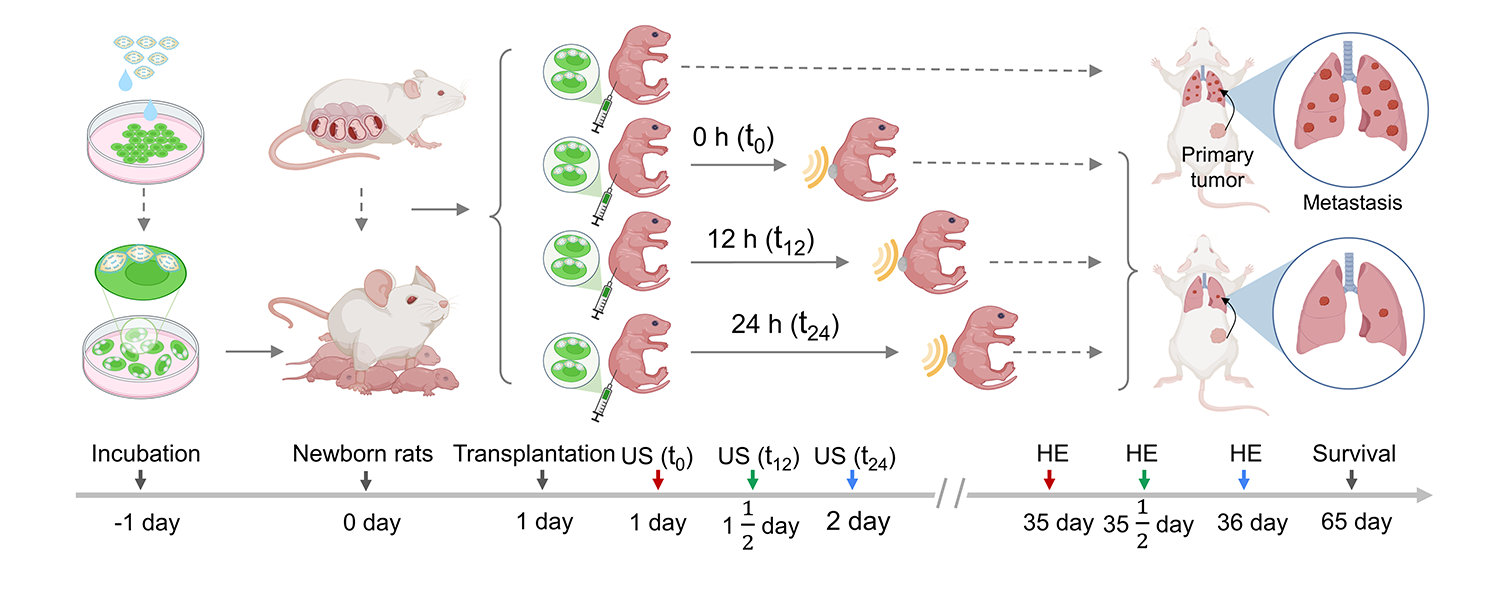


**Fig.S10. Intracellular cavitation-mediated delivery of E-cadherin inhibited tumor metastasis *in vivo*.**

Schematic illustration of the delivery of the nuclear *E-cadherin* gene in the C6 cells of the subcutaneously transplanted neonatal rat model. Created with BioRender.

**Fig.S11.**


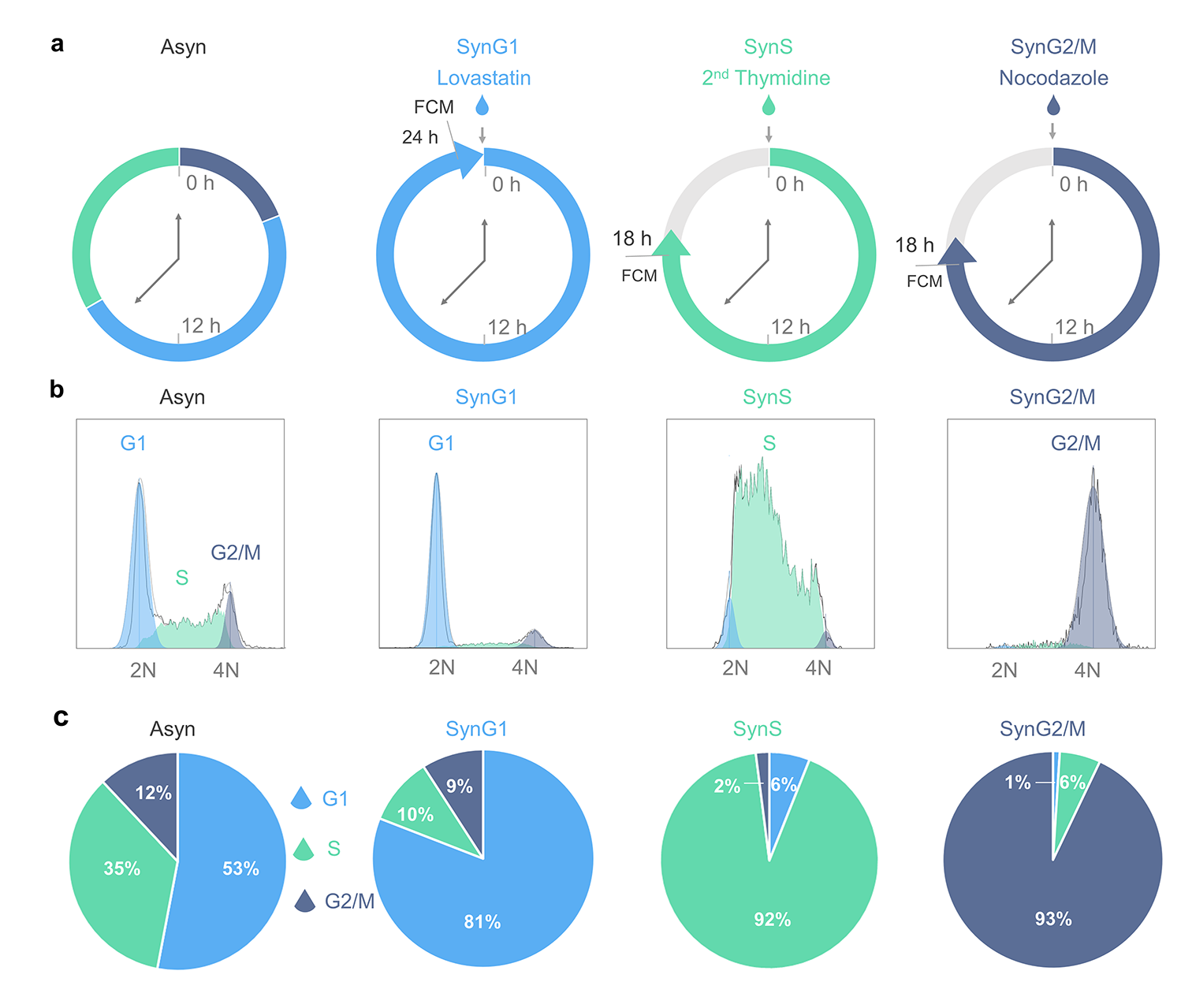


**Fig.S11. Cell cycle synchronization analysis by flow cytometry.**

**a** Schematic illustration of the cell cycle synchronization procedure. C6 cells were cultured in a medium supplied with lovastatin for the G1 phase, thymidine for the S phase or nocodazole for the G2/M phase. The time was needed for the G1, S or G2/M phases was different. **b**, Representative flow cytometry histograms of synchronized C6 cells, indicating the distribution of DNA content in C6 cells in the asynchronized phase (Asyn), synchronized in the G1 phase (SynG1), synchronized in the S phase (SynS) or synchronized in the G2/M phase (Syn G2/M) phases. **c**, Percentage of cells in the G1, S, or G2/M phases for these asynchronized or synchronized C6 cells.

**Fig.S12.**


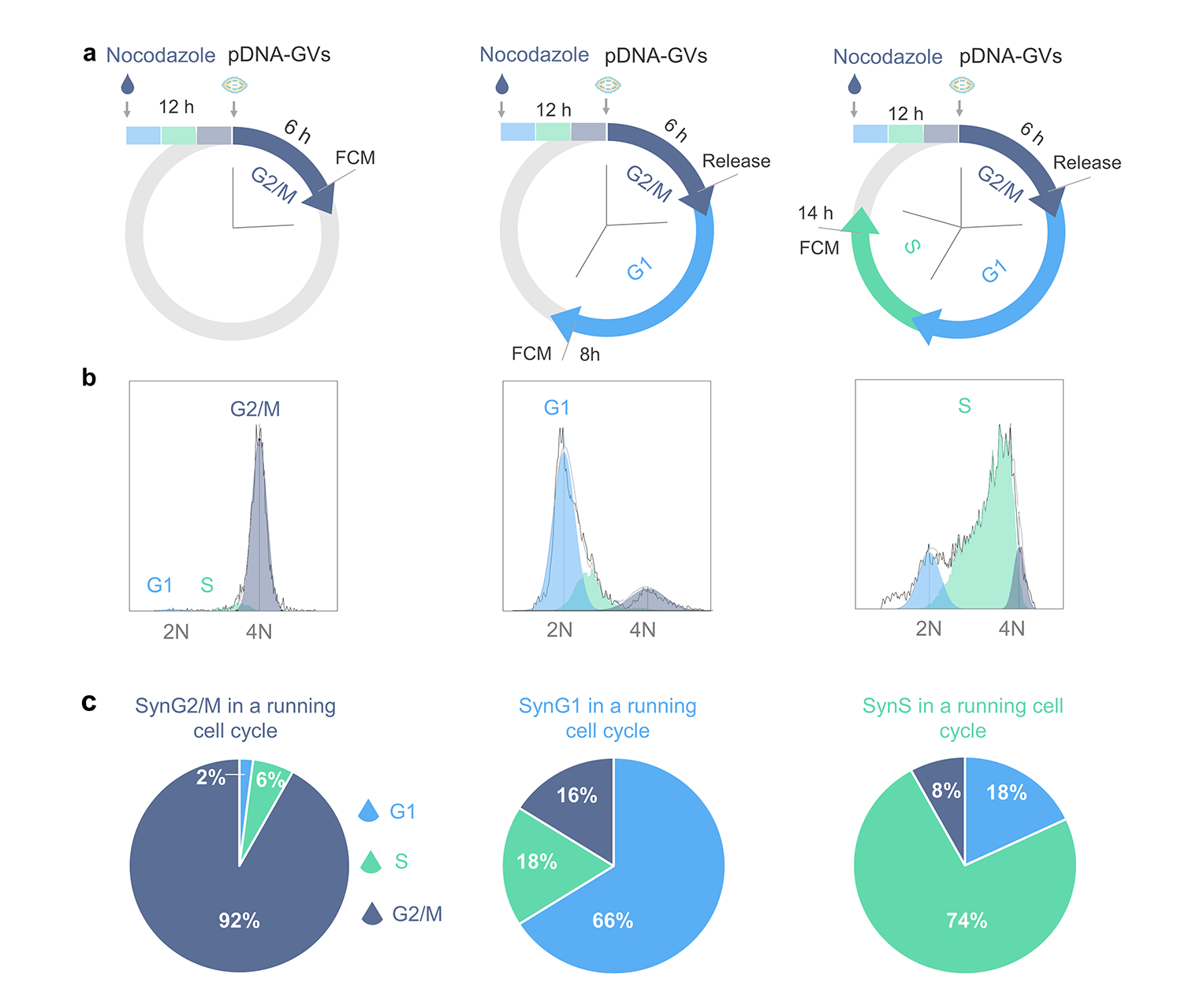


**Fig.S12. Acoustic control of nuclear gene delivery in a cell cycle running from the G2/M phase.**

**a** Schematic illustration of the cell synchronization and nuclear gene delivery procedure. Cells were first synchronized with nocodazole for 12 h, and then, pDNA-GVs were added for intracellular uptake for another 6 h. After being rinsed with PBS, these synchronized pDNA-GVs@C6 cells were released in nocodazole-free medium and reentry into the cell cycle from G2/M. These cells in a running cell cycle at 0 h, 8 h or 14 h after release were collected for flow cytometry analysis. **b**, Representative flow cytometric histograms of these pDNA-GVs@C6 cells, indicating the distribution of DNA content in the SynG2/M, SynG1 or SynS phases in a running cell cycle. **c**, Percentage of cells in the G1, S, or G2/M phases.

**Fig.S13.**


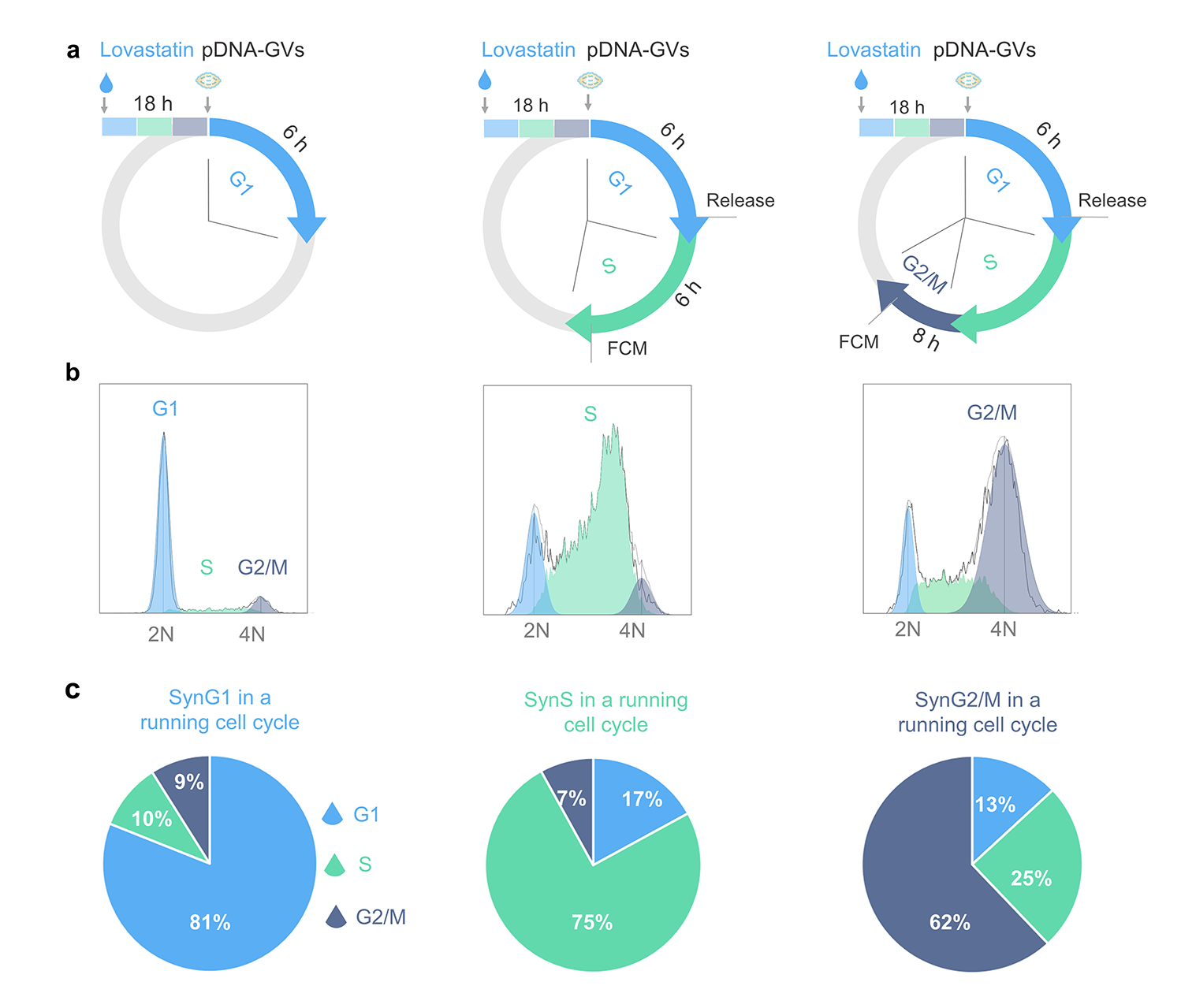


**Fig.S13. Acoustic control of nuclear gene delivery in a running cell cycle from the G1 phase.**

**a** Schematic illustration of the cell synchronization and nuclear gene delivery procedure. Cells were first synchronized with lovastatin for 18 h, and then, pDNA-GVs were added for uptake for another 6 h. After being rinsed with PBS, these synchronized pDNA-GVs@C6 cells were released in a lovastatin-free medium and reentered the cell cycle from the G2/M phase. These cells in a running cell cycle at 0 h, 6 h or 8 h after release were collected for flow cytometry analysis. **b**, Representative flow cytometric histograms of these pDNA-GVs@C6 cells, indicating the distribution of DNA content in the SynG2/M, SynG1 or SynS phases in a running cell cycle. **c**, Percentage of cells in the G1, S, or G2/M phases.

**Fig.S14.**


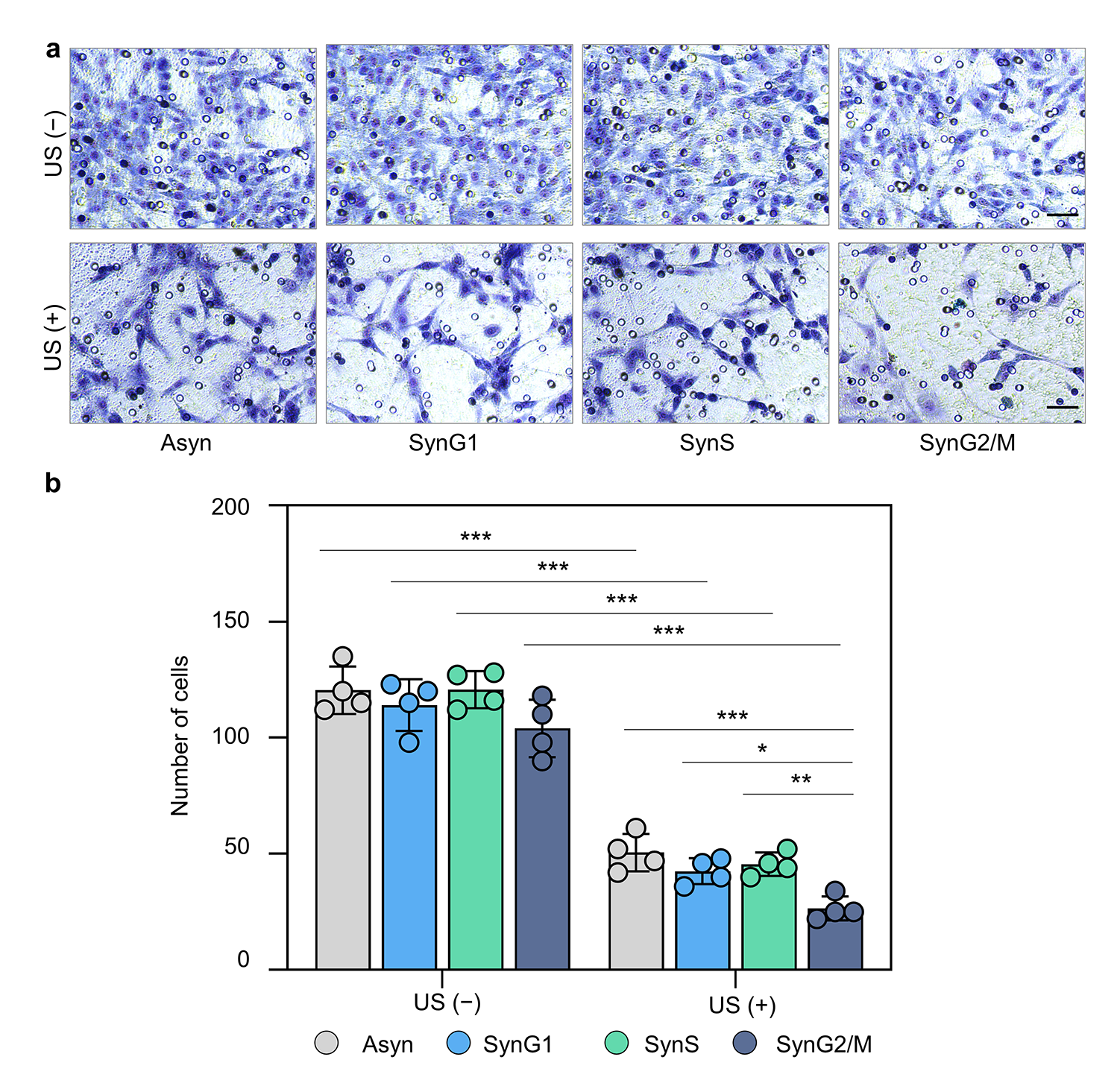


**Fig.S14.** **Cell cycle-dependent nuclear *E-cadherin* delivery enhanced inhibition of tumor cell invasion.**

**a**, Representative images of the Transwell assay for synchronized or synchronized pEcad-GVs@C6 cells treated with or without acoustic irradiation in a running cell cycle from the G1 phase. Ultrasound was applied to asynchronized pEcad-GVs@C6 cells and synchronized pEcad-GVs@C6 cells in the G2/M, G1, or S phrases in the running cell cycle. Scale bar = 25 µm. **b**, The quantitative results of Fig.a showed that a stronger inhibitory effect of tumor cell migration when nuclear *E-cadherin* delivery was applied in the G1 phase in a running cell cycle. n = 4. *P<0.05, **P<0.01, ***P<0.001.

**Fig.S15.**


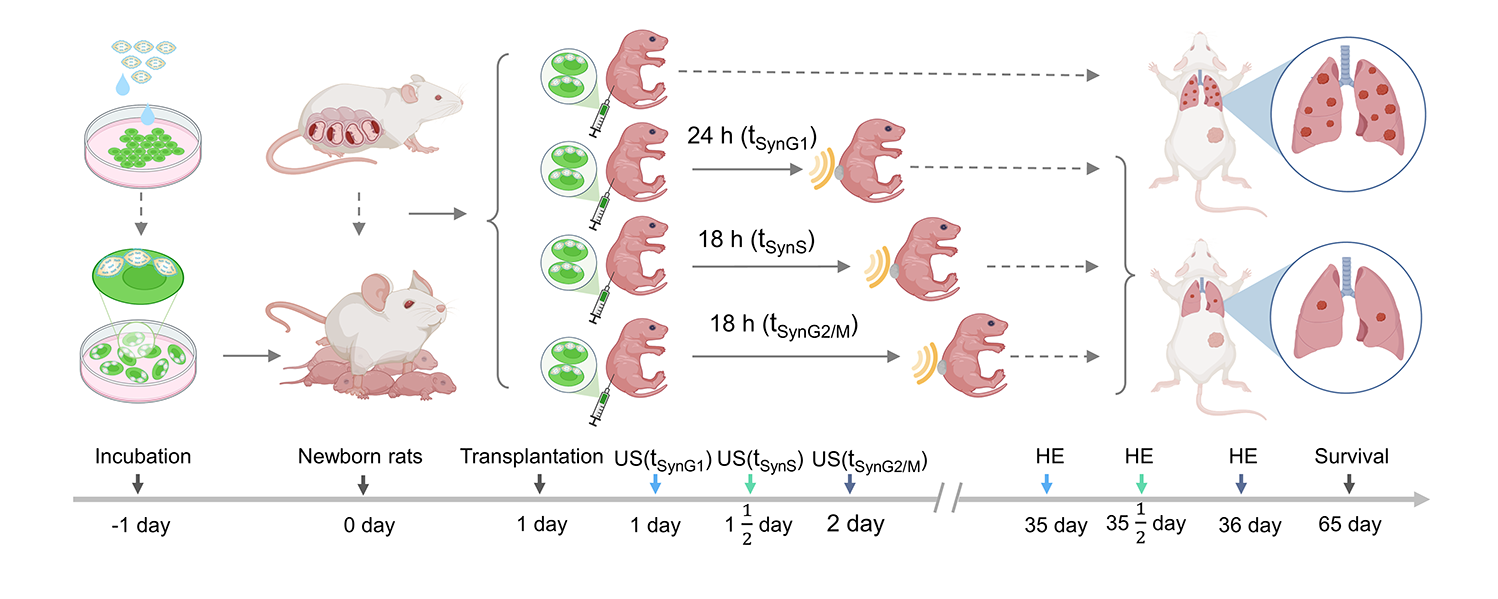


**Fig.S15.** ***In vivo* cell cycle-dependent nuclear gene delivery.**

Schematic illustration of nuclear delivery of the *E-cadherin* gene in the C6 subcutaneously transplanted neonatal rat model at different cell cycle phases. t_SynG1_, t_SynS_ and t_SynG2/M_ stand for the synchronized pEcad-GVs@C6 cells that were treated with acoustic irradiation in the G1, S, or G2/M phases in the cell cycle. Created with BioRender.

**Fig.S16.**


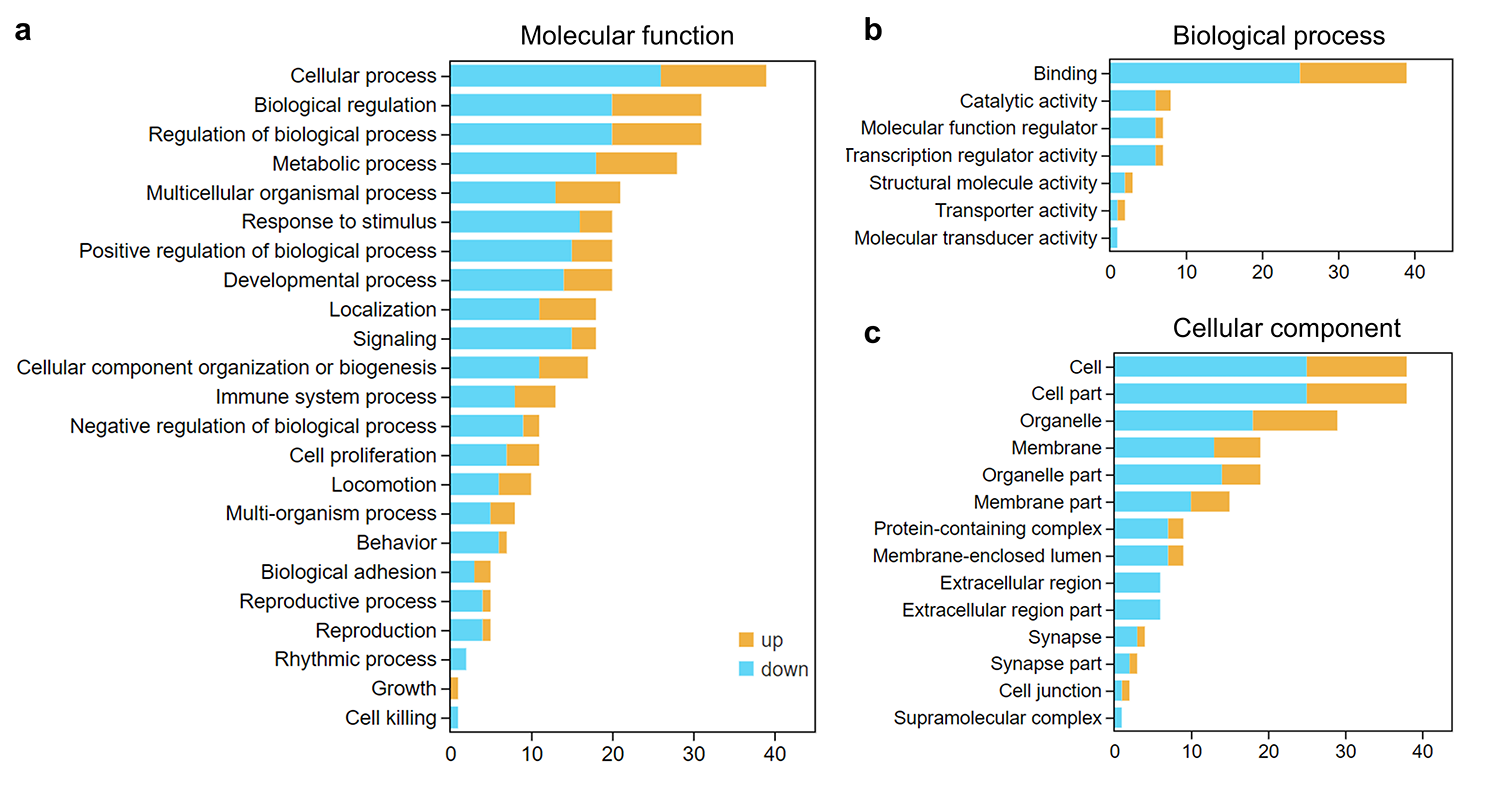


**Fig.S16.** **RNA-seq of the pEcad-GVs@C6 cells**.

**a-c,** Synchronized pCherry-GVs@C6 cells or pEcad-GVs@C6 cells were received with acoustic irradiation at the G2/M phase. Transfection-positive cells with mCherry fluorescence were sorted by fluorescence-activated cell sorting (FACS) after 48 h. GO pathway analysis of RNA-seq for pCherry-GVs@C6 cells and pEcad-GVs@C6 cells received with acoustic irradiation in the SynG2/M phase.

**Fig.S17.**


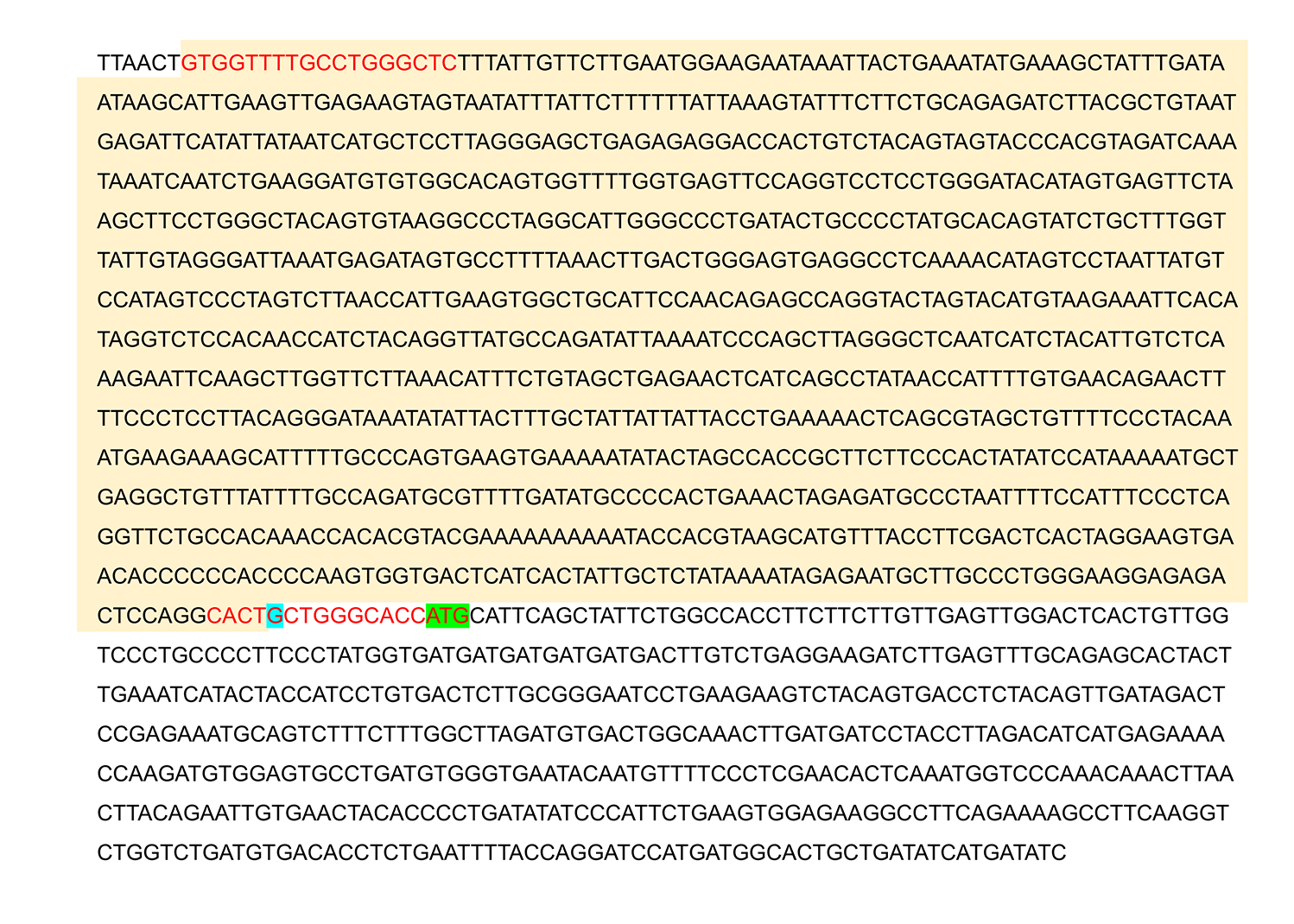


**Fig.S17.** **The promoter sequence of MMP13**.

The promoter of MPP13 located at the –1 and –1084 positions from the transcription start site was cloned (yellow). The primers used for promoter cloning were marked in red. The transcription start site was marked in blue, and the initiation codon is marked in green.

**Fig.S18.**


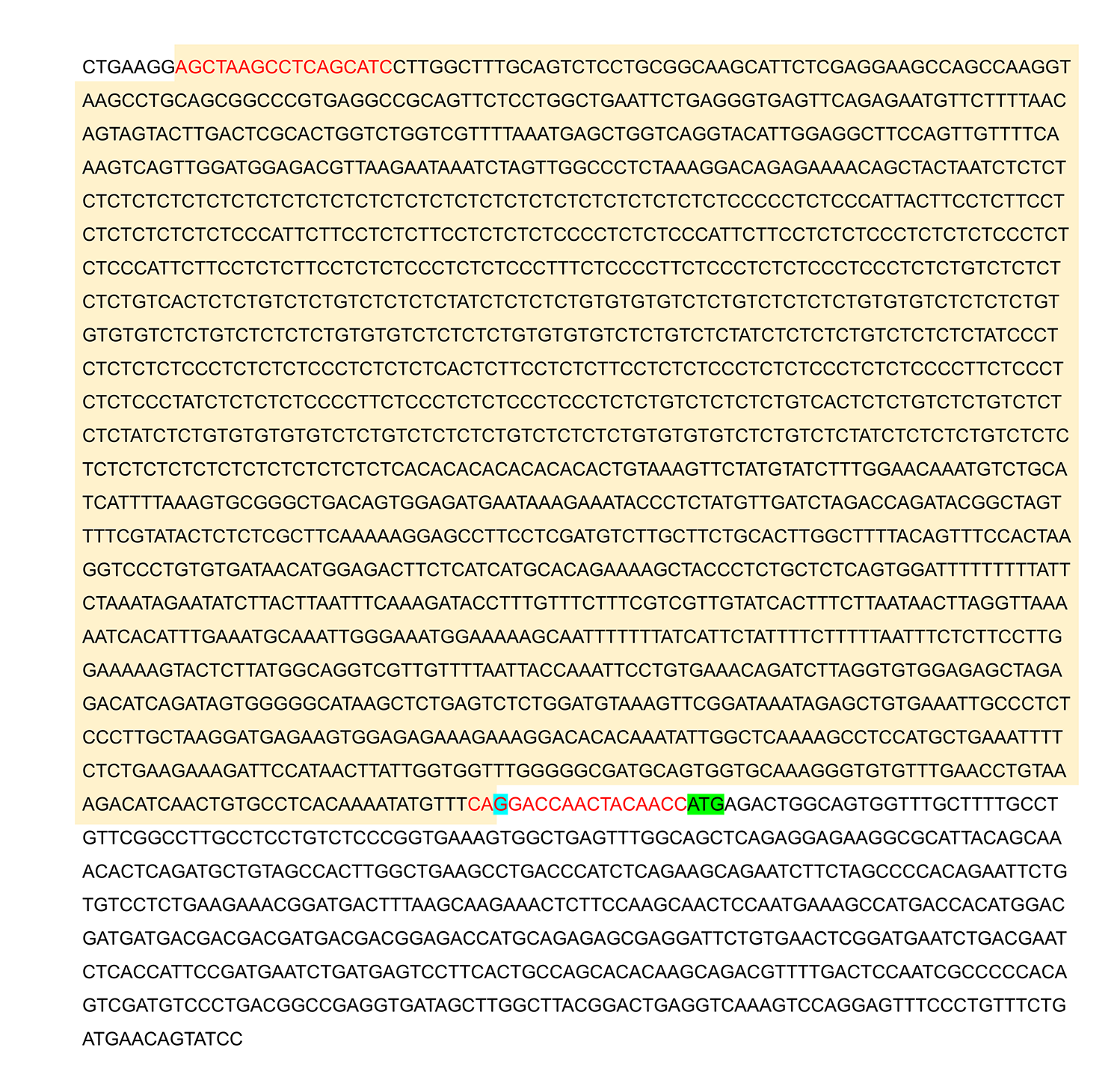


**Fig.S18. The promoter sequence of SPP1.**

The promoter of SPP1 located at the –1 and –1749 positions from the transcription start site was cloned (shown in yellow). The primers used for promoter cloning were marked in red. The transcription start site was marked in blue, and the initiation codon is marked in green.

**Fig.S19.**


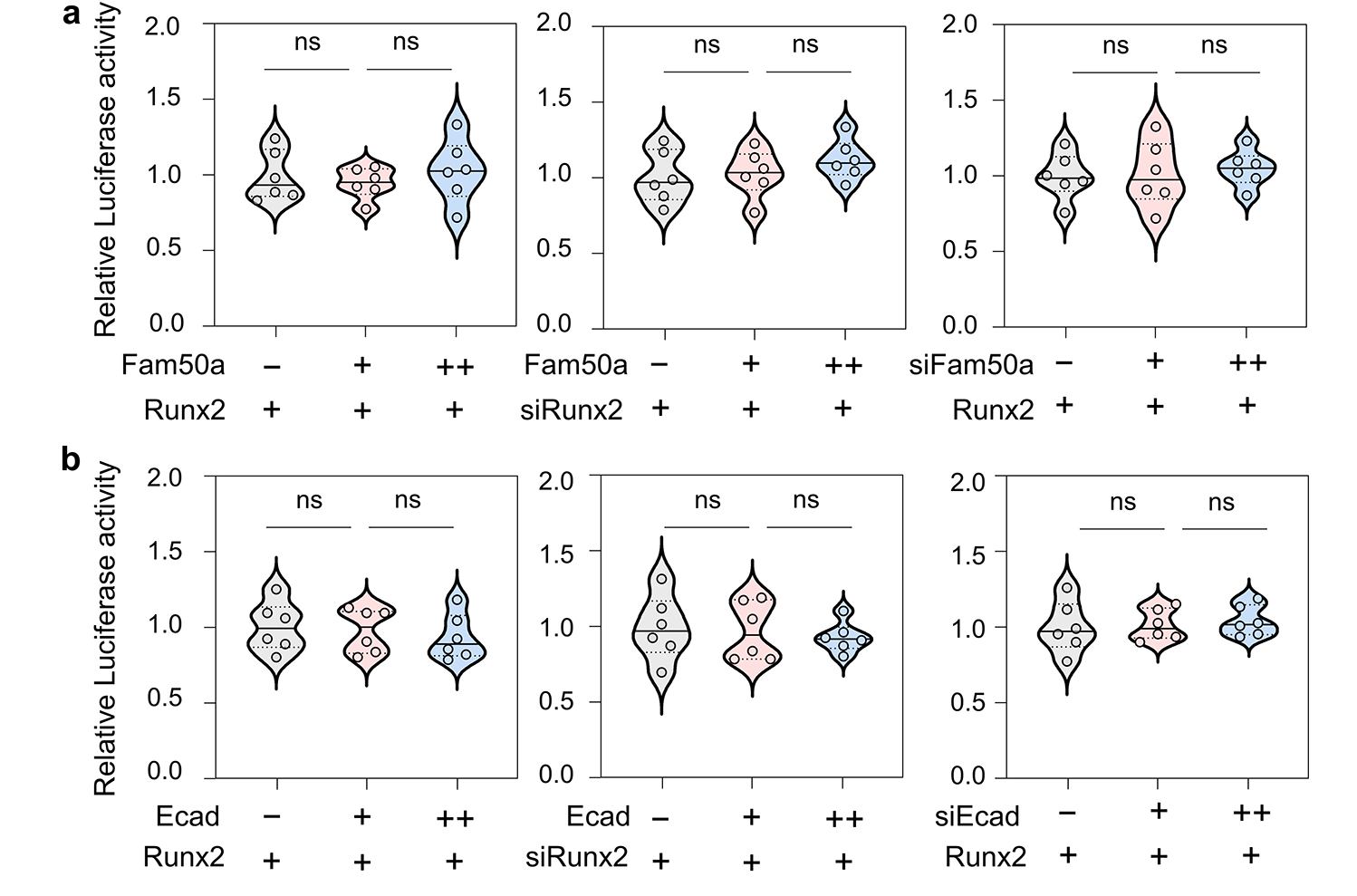


**Fig.S19.** **SPP1 promoter luciferase reporter assay**.

**a,** SPP1 promoter transcription activity assay in the Runx2-overexpressed or Runx2-knockdown cells transfected with different doses of Fam50a plasmids or siFam50a. n = 6. *P<0.05. n = 6. *P<0.05. **b**, SPP1 promoter transcription activity assay in the Runx2-overexpressed or Runx2-knockdown cells transfected with different doses of E-cadherin plasmids or siE-cadherin. n = 6. *P<0.05.

**Supplementary Tables**

**Table S1.**

**Forward and reverse primer sequences used for real-time PCR.**

| **mRNA** | **Sequence** |  |
| --- | --- | --- |
| GAPDH | Forward | 5’-CAACTCCCTCAAGATTGTCAGCAA-3’ |
|  | Reverse | 5’-GGCATGGACTGTGGTCATGA-3’ |
| E-cadherin (CDH1) | Forward | 5’-GAGGCCAAGCAGCAGTACATT-3’ |
|  | Reverse | 5’-TCTCCAGACCCACACCAAAGT-3’ |
| N-cadherin (CDH2) | Forward | 5’-TGCCCCAAATCCTAAAATCAT -3’ |
|  | Reverse | 5’-ACTCTCTGTCCAGAACAGCGA -3’ |
| MMP9 | Forward | 5’-CATGAAGACGACATAAAAGGC -3’ |
|  | Reverse | 5’-GGACACATAGTGGGAGGAGCT -3’ |
| Snail1 | Forward | 5’-CCGATGAGGACAGTGGCAAAA -3’ |
|  | Reverse | 5’-AAGCCCGGGAAGGCAATGAAG -3’ |

**Table S2.**

**Sense and Antisense sequence used for siRNA.**

| **siRNA** | **Sequence** |  |
| --- | --- | --- |
| GAPDH Positive control | Sense | 5’-UGACCUCAACUACAUGGUUTT-3’ |
|  | Antisense | 5’-AACCAUGUAGUUGAGGUCATT-3’ |
| GAPDH Negative control | Sense | 5’-UUCUCCGAACGUGUCACGUTT-3’ |
|  | Antisense | 5’-ACGUGACACGUUCGGAGAATT-3’ |
| E-cadherin (CDH1) | Sense | 5’-GAUGACAUAAACACCUACATT-3’ |
|  | Antisense | 5’-UGUAGGUGUUUAUGUCAUCTT-3’ |
| Fam50a | Sense | 5’-GGAGAAUCGGCUUCGGGAATT-3’ |
|  | Antisense | 5’-UUCCCGAAGCCGAUUCUCCTT-3’ |
| Runx2 | Sense | 5’-GCACUCCAUAUCUCUACUATT-3’ |
|  | Antisense | 5’-UAGUAGAGAUAUGGAGUGCTT-3’ |
